# Machine Learning and Deep Learning in Synthetic Biology: Key Architectures, Applications, and Challenges

**DOI:** 10.1021/acsomega.3c05913

**Published:** 2024-02-19

**Authors:** Manoj Kumar Goshisht

**Affiliations:** Department of Chemistry, Natural and Applied Sciences, University of Wisconsin—Green Bay, Green Bay, Wisconsin 54311-7001, United States

## Abstract

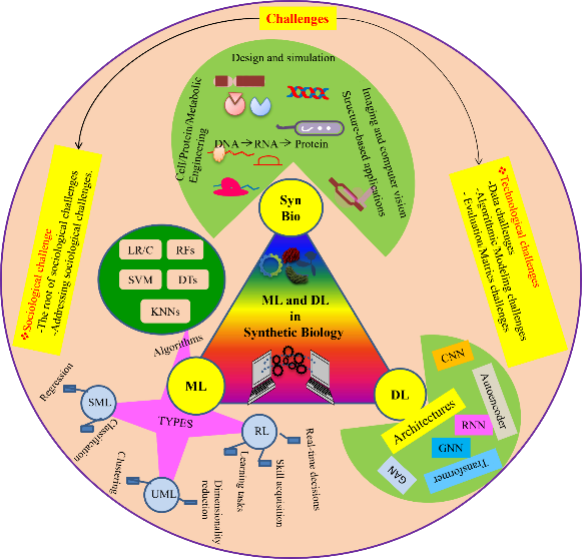

Machine learning (ML), particularly deep learning (DL), has made rapid and substantial progress in synthetic biology in recent years. Biotechnological applications of biosystems, including pathways, enzymes, and whole cells, are being probed frequently with time. The intricacy and interconnectedness of biosystems make it challenging to design them with the desired properties. ML and DL have a synergy with synthetic biology. Synthetic biology can be employed to produce large data sets for training models (for instance, by utilizing DNA synthesis), and ML/DL models can be employed to inform design (for example, by generating new parts or advising unrivaled experiments to perform). This potential has recently been brought to light by research at the intersection of engineering biology and ML/DL through achievements like the design of novel biological components, best experimental design, automated analysis of microscopy data, protein structure prediction, and biomolecular implementations of ANNs (Artificial Neural Networks). I have divided this review into three sections. In the first section, I describe predictive potential and basics of ML along with myriad applications in synthetic biology, especially in engineering cells, activity of proteins, and metabolic pathways. In the second section, I describe fundamental DL architectures and their applications in synthetic biology. Finally, I describe different challenges causing hurdles in the progress of ML/DL and synthetic biology along with their solutions.

## Introduction

Over the past two decades, biology has undergone a massive transformation that makes it possible to effectively build biological systems. The fundamental force behind this abrupt transition is the genomic revolution,^1^ which made it possible to sequence the DNA of a cell. With CRISPR-based technologies,^2^ it is now possible to accurately modify DNA in vivo, which is among the newest advances and techniques made possible by this genomic revolution. Precision DNA editing and high-throughput phenotypic data offer an exciting opportunity to connect phenotypic alterations to underlying code modifications. The goal of synthetic biology is to develop biological systems that meet specific requirements,^3^ for instance, cells responding in a particular way to external stimuli or generating the requisite quantity of biofuel. To achieve this, synthetic biologists make use of engineering design concepts to employ engineering’s predictability to regulate intricate biological systems. Standardized genetic components and the Design–Build–Test–Learn (DBTL) cycle are two examples of engineering approaches that are applied iteratively to get the desired result. According to the synthetic biology DBTL cycle, this discipline goes through the following four stages: (i) *Design*: Conjecture a DNA pattern or series of cellular alterations that can accomplish specified objectives of the plan. (ii) *Build*: This mainly entails the development of the DNA fragment and its effective incorporation into a cell. (iii) *Test*: Provide data to determine how well the assessed phenotype reaches the desired outcome and assesses the impact of off targeted or unintended effects. (iv) *Learn*: Use the test data to discover principles that direct the cycle toward the desired outcomes more effectively than a random search might. It frequently involves identifying errors that result from unintended off-target impacts. Modification to a pathway can result in a flux redistribution leading to byproducts, toxicity, slower cell growth, or several other outcomes that must be addressed. The next set of designs can be guided by artificial intelligence (AI), which would decrease the number of DBTL repetitions required to attain the desired result. Synthetic biology generally entails genomic alterations to urge a cell to produce products or behave in a specific manner.

ML has come to light as a promising option to speed up the progress in synthetic biology design by uncovering patterns in the data-rich accomplishments provided by systems biology. DL generally employs representations with numerous layers of artificial neurons to discover the link between the inputs and outputs. Examples comprise frameworks that use sequence information to predict the activation of components like promoters or precise protein structure forecasting algorithms.^4−7^ One of the main characteristics of DL models is their ability to gradually extract insights from input data by systematically transmitting information between layers of an artificial neural network (ANNs).^8^ For example, early layers of the network may retrieve low-level properties like vertical or horizontal edges when examining a microscope image, while the subsequent layers combine this data to determine the shape or patterns of cells in the image.^9,10^ DL networks can also encode intricate nonlinear connections between input values. For instance, a DL model that infers a protein’s function from its amino acid sequence can discover that specific combinations of amino acids operate synergistically to increase activity above what would be predicted based on the individual amino acids’ contributions.^11^

There are various obstacles that must be solved to advance synthetic biology and DL in the future. Synthetic biologists are not taught DL techniques typically; therefore, it might be challenging to keep up with two fields that are expanding quickly at the same time. Moreover, synthetic biology data sets have discipline-specific limitations. Natural sequence information is one area where there is a wealth of data, but the diversity of these data sets is constrained since nonfunctional patterns or those that have high levels of expression are often underrepresented. As a result of practical limitations in the execution and evaluation of synthetic biology components, the quantity of information available for other applications is greatly limited.

**Figure 1:**
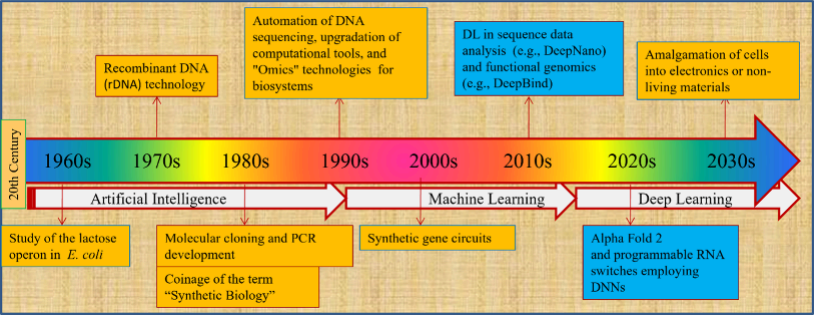
An overview of the advances in ML/DL and synthetic biology since the 1960s.

This review seeks to assist synthetic biologists in comprehending and applying ML and DL strategies in their research by presenting an overview of techniques and summarizing recent advances at the nexus of ML/DL and engineering biology (Figure 1). I begin by describing barriers in the progress of synthetic biology and the predictive potential of ML in overcoming these barriers. Then, I have described ML scenarios, mathematical frameworks, and their applicability in cell, protein, and metabolic engineering. Afterward, I review prevalent DL network architectures pertinent to engineering biology applications. Next, I describe recent advances that leverage DL to enable synthetic biology, emphasizing examples from component design, imaging, structure-based learning, and other fields. Finally, I present challenges pertinent to ML, DL, and synthetic biology and their possible solutions.

## Predictive Potential of ML

By learning the basic pattern in experimental results, machine learning can give predictive power without the requirement of complete mechanistic insight. Training data is employed to statistically relate a set of inputs to a set of outputs using sufficiently expressive models that reflect practically any relationship and is free from assumptions in prior knowledge. Machine learning has been applied in this context to forecast pathway dynamics, tune pathways via translational control, detect cancers in breast tissues, diagnose skin cancer, and determine RNA and DNA protein-binding motifs.^12−14^ Moreover, machine learning can be utilized to create synthetic biology systems by understanding the connection between phenotype and the genetic parts employed in genetic circuits, allowing for more stable circuits. However, ML algorithms are data hungry. They require a large amount of data to be trained and be efficacious. The recent machine learning revolution was enabled not by new techniques but by (i) increasing computational power and (ii) the accessibility of massive training libraries.^15,16^ Artificial vision would have probably not extended superhuman performance if it had to be taught on pictures taken on photographic film and mailed physically from photographers to AI researchers. The accessibility of vast image libraries facilitated by automated digital image collecting using charge-coupled device (CCD) cameras, as well as their distribution via the Internet, has been vital to its advancement.

## Categories of Ml Methods

ML is an AI subset that enables computers to acquire knowledge from experience. ML algorithms employ computational approaches to “learn” particulars directly from data without depending on a preordained equation as a representation. The ML algorithms advance their performance adaptively in the presence of excess samples available for learning. In general, the more the training data, the more accurate and precise the learned function. Tens of thousands of ML algorithms exist, and hundreds of new ones are developed annually. When creating an ML model, input representation, loss function, output variables, hyperparameters, and model evaluation are significant considerations. The types of ML are described below in brief.

### Supervised Machine Learning (SML)

**Figure 2:**
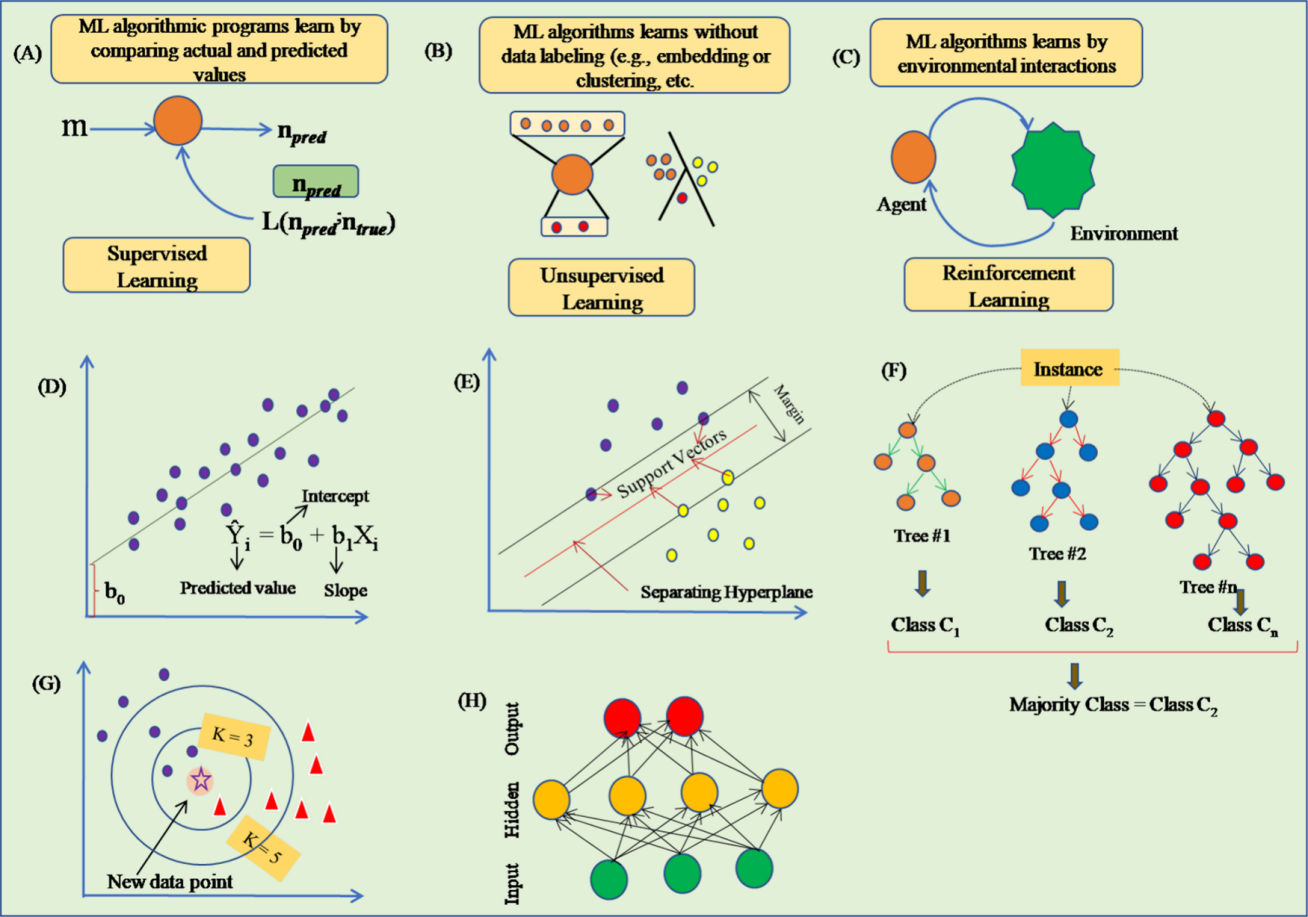
Schematic representation of machine learning scenarios and mathematical frameworks. (A) SML in which data sets involve ground truth labels. (B) UML in which data sets do not involve ground truth labels. (C) Reinforcement learning where interaction between an algorithmic agent and simulated environment takes place. (D) Linear regression/classification that can be employed to fit models in which the output is a scalar value and data can be predicted by a straight line. (E) Support vector machines locate a separating hyper-plane that parts data into classes. (F) RFs employ the “bagging” technique to construct complete decision trees (DTs) in parallel using random bootstrap instances of the data sets and attributes. RFs select the most labels between different randomized DTs. (G) k-NN is employed for both regression as well as classification, and the input comprises the *k* nearest training instances in the data set. The output relies on whether the *k*-NN is employed for regression or classification. (H) NNs generally form a feedforward network of weights in which inputs trigger the hidden layers which give output. However, NNs also form a feedback network in which NNs learn by back-propagation through the networks.

SML is the most fundamental type of ML in which an algorithm is instructed on the labeled data. SML methods identify patterns of correlation between input attributes and output variables. The objective is to learn a task that perfectly delineates the relationship between the input attributes and output value in labeled data. Generally, there is direct a relation between the training data and the accuracy of learned tasks, however, the entailed size of training data also relies upon the attributes employed for the specific task. This solution is subsequently deployed for usage with the final data set, from which it learns in the same way as it learned from the training data set. In regression type, an output label is real-valued continuous variables whereas in classification type, the output label is a discrete variable (Figure 2A).

### Unsupervised Machine Learning (UML)

UML has the advantage of working with unlabeled data. The algorithms employ clustering approaches, clustering data points with identical attributes into prominent features with little information loss. Hence, the appraisal generally depends on fact-finding analysis. These algorithms attempt to apply approaches to the input data to explore for rules, find patterns, summarize and cluster data points, derive useful insights, and better communicate the data to users (Figure 2B). For more details on SML and UML, I refer the readers to an ML-based book.^17^

### Reinforcement Learning (RL)

RL is directly inspired by how humans learn from events in their daily lives. It has an algorithm that uses trial and error to better itself and learn from new scenarios. Favorable outputs are rewarded, and nonfavorable outputs are rejected. Reinforcement learning, which is built on the psychological idea of conditioning, works by setting the algorithm in a workplace setting with an interpreter and rewards. The output result is delivered to the interpreter at every algorithmic iteration, which decides if the outcome is beneficial or not. If the result is favorable, the interpreter reinforces it by rewarding the algorithm whereas, in case of unfavorable results, the algorithm is compelled to repeat until a better result is found. Generally, the reward system is closely related to the efficacy of the outcome. Due to the availability of large training data sets from simulations under various genetic settings, RL algorithms can provide an efficient computational method to aid in decision-making in the DBTL cycle (Figure 2C).

### Semisupervised Machine Learning (SSML)

By employing small labeled and large unlabeled data sets, SSML boosts the efficiency of a supervised model. It can reduce the requirement for vast amounts of organized and human-labeled data along with filtering the systemic noise arising in biological measurements due to various experimental variables. Because SSML is compatible with small training sets, it may have considerable potential in organisms, particularly metazoans with fewer experiment-aided genetic interactive gene pairs.

### Active Learning (AL)

AL is a special case of SML. This method is used to create an effective classifier while minimizing the amount of the training data set by actively organizing the valuable data points.

### Transfer Learning (TL)

Standard ML approaches presume that the training and testing contexts have the same probability distribution. This assumption, however, does not hold in the situation of merging biological data from several platforms. TL refers to the situation when a classifier is trained on one data set and then tested on another data set that may have a completely diverse probability distribution function. Biological data produced from several platforms and maybe employing various technologies is an obvious option for transfer learning approaches. For example, features acquired from the prediction of yeast growth rate may be transferred to other predictive tasks,^18^ including predicting ethanol generation in yeast.

## Common Ml Algorithms Used in Synthetic Biology

In this section, I discuss a few specific algorithms employed in synthetic biology applications.

### Linear Regression or Classification

The linear regression algorithm^19^ is based on SML. It carries out a regression task. In this algorithm, a linear equation is used to simulate the connection between inputs and outputs. Linear models are simple to design and analyze, but the connection between the objective variable and the attribute in several applications extends more than a linear function. However, linear regression is not appropriate for classification since it concerns continuous values, while classification issues require discrete values. The second issue is the shifting in threshold value caused by the addition of new data points (Figure 2D).

### Support Vector Machines (SVMs)

Several researchers prefer SVM^20^ because it produces substantial accuracy while using minimal computing power. SVM is useful for both classification and regression tasks. Nonetheless, it is commonly employed in classification tasks. The SVM algorithm learns a collection of ideal hyperplanes that can classify samples. For each class, it maximizes the distance between the hyperplane and the closest data point. The data points (support vectors) assist in developing SVM. Increasing the margin distance gives some reinforcement, allowing future data points to be classified with greater certainty. Soft margin SVMs encompass “slack” variables that permit a few data points to be incorrectly categorized and are effective when data is not differentiable (Figure 2E).

### Random Forests (RFs)

Random forest^21^ is a popular ML technique that integrates the output of numerous decision trees to produce a single conclusion. Its ease of usage, flexibility, and ability to tackle classification and regression challenges have boosted its popularity. The RF model is composed of several decision trees (DTs). While DTs are popular SML algorithms, they might suffer from bias and overfitting. When numerous DTs create an ensemble in the RF algorithm, the results are more accurate when the individual trees are not correlated with one another. The RF algorithm is a bagging method extension that employs both bagging and feature randomization to produce an uncorrelated forest of DTs. DTs build tree-like classifiers by progressively splitting data about specific attributes, most frequently employing classification performance to determine which trait and value to split (Figure 2F). RF techniques have three major hyperparameters that must be regulated before training. These hyperparameters include node size, number of attributes sampled, and number of trees. From there, the RF classifier can be applied to address regression or classification issues.

### *k*-Nearest Neighbors

The *k*-nearest neighbors (KNNs)^22^ technique is a straightforward SML approach that can be used to address classification and regression issues. However, it is mostly employed to solve classification difficulties. Most SML methods use training data to learn a task and predict unknown data, while NNs preserve the training data and the pairing distances between them to classify unknown data points with the labels of close training data points. It is known as a lazy learner since it does not do any training when given training data. Instead, it simply saves the information during the training period and makes no calculations. It does not create a model until a query is run on the data set. As a result, KNN is significant for data mining. Here, “K” refers to the number of nearest neighbors employed for predicting unknown points (Figure 2G).

### Neural Networks

Neural networks (NNs),^23^ also called simulated neural networks (SNNs) or artificial neural networks (ANNs), are nonlinear statistical decision-making or data modeling tools. They can be applied to identify patterns in data or to model intricate connections among inputs and outputs. Each node in a NN, which is commonly referred to as a neuron, is connected to every other node by a link, each of which is assigned a weight and threshold. The network is referred to as feedforward when neurons are exclusively connected to other neurons in succeeding layers. On the contrary, a network is referred to as recurrent when neurons in the same layer communicate with one another. The output layer serves as the last layer that gives the model predictions, while the input layer is the first layer that receives the representations of each incident as input. Hidden layers (any layers of neurons) exist in between the input and output layers. Each neuron multiplies the input by the link weights and transforms the data using an activation function to send information to the neurons it is connected to (Figure 2H). Any node whose output exceeds the defined threshold value is activated and begins providing data to the network’s next layer. Instead, no data is transmitted to the network’s next layer. NNs depend on training data to develop and enhance their accuracy over time (Figure 2H).

## Applications of ML in Biosystems Design

The different ML approaches outlined in the preceding section stipulate a toolkit to solve the issues related to designing biological components. An ML model can be used to simulate synthetic biology applications with input and output variables that are easily quantifiable. In this section, I shall describe the assimilation of machine learning in synthetic biology, with a strong focus on cell and metabolic engineering subfields. I shall also discuss how this assimilation can help synthetic biology overcome the current difficulties in understanding the intricacies of biological systems.^24^

### Applications in Cell Engineering

Cell engineering is an area of synthetic biology that involves the assembly of biomolecules to form genetic circuits/networks that can coordinate with internal cell machinery to improve, restore, or add unique functionalities to a designated host cell.^25^ The biological components typically comprise elements that control transcription, translation, and transcriptional factors that can be utilized to control the activity of supplemental proteins.

**Figure 3:**
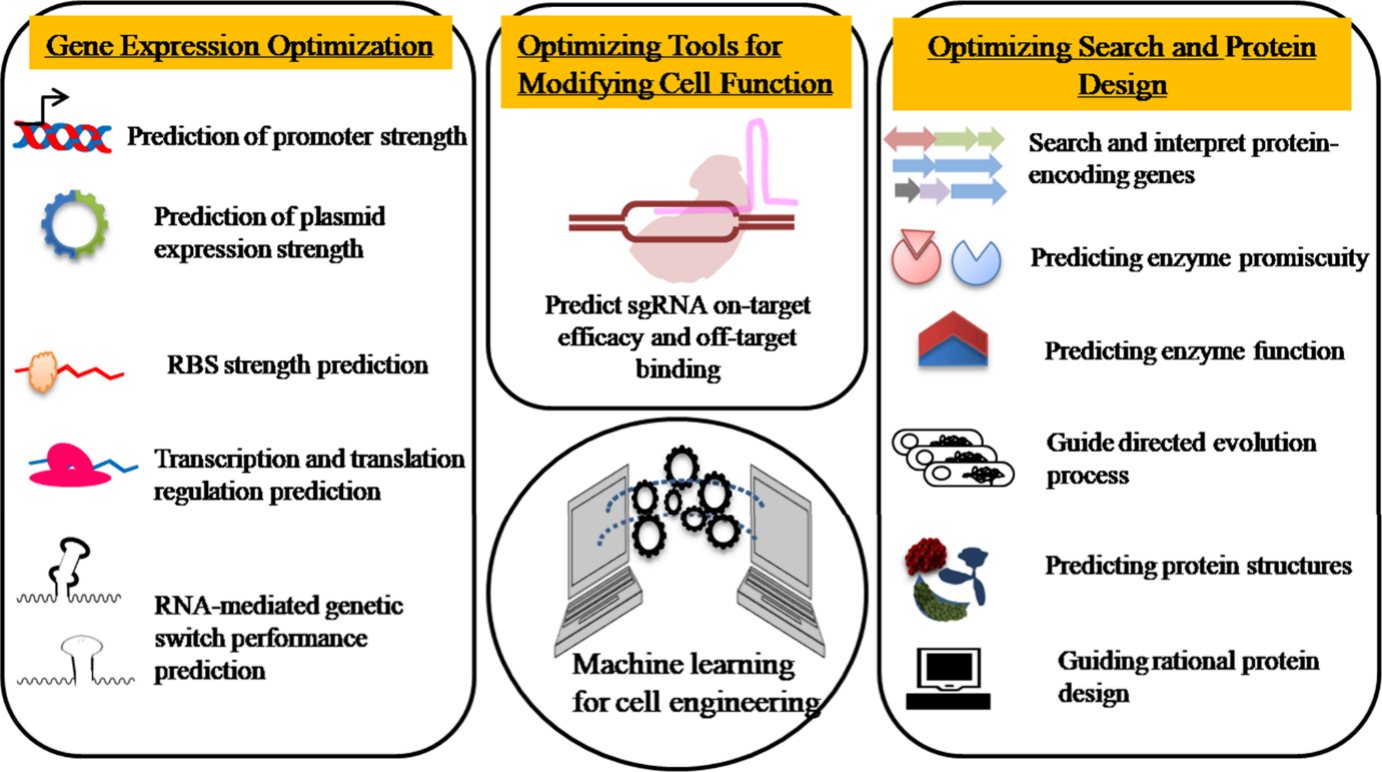
Applications of ML in cell engineering. ML can be employed for (i) improving gene expression, (ii) bettering tools for altering cellular functions, and (iii) upgrading protein search and design.

Synthetic biologists have worked to describe the performance outcomes of recognized biological components, comprehend their fundamental mode of action, and evaluate the interactions of all these components inside the host cell by trial-and-error research protocol.^26^ Although cell engineering methods have become more advanced, synthetic biologists still confront several challenges. Designing innovative biological components and discovering the interactions among host cell machinery and engineered features can be difficult due to a lack of understanding of design guidelines, causing troubleshooting issues. To that end, ML provides a way for optimally constructing and fine-tuning biomolecules in the host cell with predictable implications. It has multiple applications in gene expression optimization, cellular function modification, and protein designing (Figure 3).

Several researchers started to use neural networks to guide the data-driven design of promoters^4,27,28^ and RBS sequences^29^ for regulating gene expression. Meng et al. used neural networks to estimate promoter strength using altered promoters and RBS motifs as inputs.^30^ Interestingly, their technique outperformed even mechanistic frameworks based on position weight matrices and methods of thermodynamics.^31−33^

ML can determine gene expression by optimizing the biological modules involved in translation and transcription, in addition to promoters and RBS sequences. Tunney et al. employed a feedforward neural network architecture, in which information is continuously “fed forward” from one stratum to the next, mimicking biological processes for predicting ribosome distribution across mRNA transcripts and translation elongation speeds from mRNA transcript coding sequences.^34^ Besides the development of biological components to control gene expression, more efficient strategies for changing cell function are required. This can be accomplished by removing undesirable genes or permanently incorporating foreign biomolecules into the cell genome utilizing genome editing systems such as the CRISPR-Cas system. Even though these tools have transformed the synthetic biology field, there is still potential to optimize CRISPR-Cas tools for identifying and optimizing sgRNA binding to the intended target site while decreasing off-target binding. Previous research employed the support vector machine algorithm, a form of supervised ML, to improve CRISPR-Cas9 efficiency^35,36^ but was hampered by the small size and poor quality of training data. The integration of higher-throughput screening techniques and deep learning, on the other hand, has enhanced the efficiency of modern sgRNA activity prediction algorithms. The DeepCpf1 tool, for example, prognosticates on-target knockout efficiency (indel frequencies)^37^ using DNNs trained on vast sgRNA (AsCpf1: Cpf1 from *Acidaminococcus sp. BV3L6*) task data sets.

In cell engineering, ML can be used to identify and describe protein-encoding genes in the genome. It is beneficial for creating and constructing metabolic pathways in the production host cells.^38^ The hidden Markov model has traditionally been utilized for this purpose.^39,40^ Genes are found in the genome using protein-coding signatures such as the Shine-Dalgarno sequence and subsequently functionally annotated using a sequence homology analysis against a database of known proteins. ML might discover and detect enzymes that can catalyze new reactions via enzyme promiscuity, in addition to assessing enzyme function. Chemoinformatic methods, molecular mechanics, and partitioned quantum mechanics, for example, can be employed to envisage metabolite-protein correlations in silico.^41^ These strategies, however, are computationally complex and necessitate domain expertise. Similarly, more robust, and efficient approaches, such as the Gaussian process model^42^ and support vector machine,^43^ are increasingly being employed to explore and match promiscuous enzymes to reactions. These approaches predict protein sequences (for example, K-mers), reaction signatures (for instance, chemical transformation properties, functional groups), and protein substrate affinity (Km values). Metabolic engineers now enjoy novel approaches to finding enzymes for innovative biochemical reactions while no recognized enzyme is available. Very recently, Yu et al.^44^ presented a CLEAN (Contrastive Learning-enabled Enzyme Annotation) ML algorithm for assigning Enzyme Commission (EC) numbers to enzymes with improved reliability, sensitivity, and accuracy compared to BLASTp, which is a commonly used tool for comparing protein sequences. The key features of CLEAN include its contrastive learning framework, which enables it to perform better in several aspects like (i) annotation of understudied enzymes, (ii) identification of promiscuous enzymes, and (iii) correction of mislabeled enzymes. Hence, CLEAN appears to be a promising tool for enzyme function prediction, leveraging contrastive learning to enhance accuracy and reliability, making it valuable for researchers in diverse biological and biotechnological domains.

Another ML application involves the designing and engineering of proteins. The most prevalent method is directed evolution, in which proteins undergo repeating processes of mutation and selection until the intended function and performance are obtained.^45^ By lowering the number of experimental repetitions required to achieve the desired protein, ML can steer the directed evolutionary process. It entails using past experimental data, which includes the sequence of each protein and its functional performance, to produce a library of variants with more fitness. Wu et al. simultaneously deployed different ML models and selected the models with the maximum accuracy to effectively produce nitric oxide dioxygenase and human guanine nucleotide-binding proteins from *Rhodothermus marinus*.^46^ Machine learning-aided directed evolution has also been employed to boost enzyme output,^47^ change the colors of fluorescent proteins,^48^ and improve the thermostability of proteins.^49^

Aside from directed evolution, ML can help with rational protein design. UniRep, for example, may use neural networks to learn statistical depictions of proteins (for instance, structural, evolutionary, functional, and physicochemical properties) from 24 million UniRef50 sequences.^50^ The method could predict the stability of a vast proportion of de novo proteins as well as functional alterations caused by genetic variations in wild-type proteins. Even with a small pool of training data, Biswas et al. used UniRep to improve the design of a green fluorescent protein (GFP) from *Aequorea Victoria* jellyfish and TEM-1-lactamase enzyme from *E. coli*.^51^ Another study employed neural networks that had been trained to correlate amino acids with the spatial orientation of oxygen, carbon, sulfur, and nitrogen atoms within a protein. The researchers succeeded in recognizing unique gain-of-function mutations and enhancing the protein function of three separate proteins.^52,53^

### Applications in Metabolic Engineering

Rather than designing and regulating the synthesis of a single protein and single gene expression, the subfield entails rebuilding pathways that affect the engineered organism’s metabolism. Metabolic engineering entails changing cells’ natural chemical interactions to focus on generating desired biological molecules. It is typically a multistep process that involves multiple enzymes. While the cells can synthesize various enzyme pathways and specific products, they usually require a small group of ubiquitous metabolites or cofactors.^54^ Hence, while attempting to maximize the yields of a particular metabolite, it is vital to consider the overall cellular state of affairs.^55^ A single compound, for example, could be a result of several metabolic pathways.^56^ While high-yield pathways have been built via rational design,^57−59^ these efforts are most effective for simple pathways and necessitate extensive knowledge of the enzyme processes entailed and significant experimental expertise.

**Figure 4:**
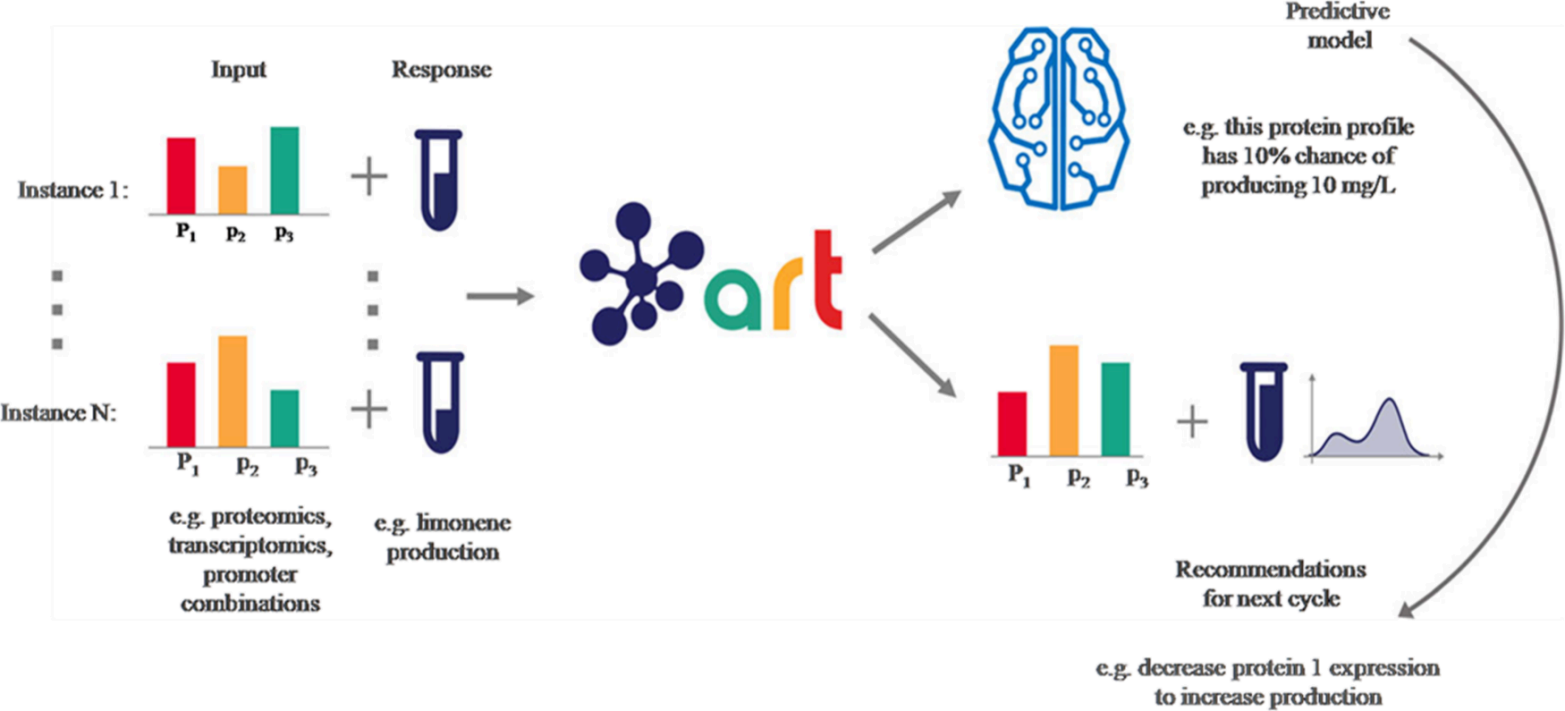
ART gives predictions and recommendations for the following cycle. ART employs experimental data for (i) constructing a probable predictive representation that predicts response from input variables and (ii) utilizes this model to give a set of recommended inputs for the following experiment that will assist in reaching the desired goal. The predicted response for the directed inputs is specified as an entire probability distribution, efficiently quantifying unpredictability. Instances have relevance to each of the diverse examples of input and response employed for training the algorithm. Reproduced with permission from ref (54). (Licensed under a Creative Commons Attribution 4.0: http://creativecommons.org/licenses/by/4.0/). Copyright 2020, Radivojević et al. Nature Research.

One big problem for ML in metabolic engineering is producing large biological data sets for training algorithms. To address this constraint, Radivojevic et al. created automated recommendation tool (ART), a machine-learning tool that combines network optimization with experimental design.^54^ The team achieved predictive modeling using 19 constructed strains in a test cycle by recommending experiment strategies to fulfill the desired aim. To summarize, ART provides a technology designed specifically for the demands of synthetic biologists to use the power of ML to facilitate predictable biology (Figure 4). By enabling successful inverse design, this combination of synthetic biology, ML, and automation has the potential to transform bioengineering.^55−57^

Despite their fundamentally distinct foundations, there is growing interest in combining mechanistic modeling with ML. In general, this takes advantage of the benefits of both methodologies to deliver data-driven forecasts and deep insight into the underlying biology. Imposing model limitations based on biological settings, for example, have been demonstrated to improve prediction accuracy by ignoring biologically implausible solution spaces.^58^

One avenue being investigated is the use of data derived from mechanistic representations as input for ML. Because complete genome sequences are now available, genome-scale models (GEMs) have gained favor as an engineering tool for forecasting system-wide events. GEMs are constructed from the ground up, based on stoichiometry and mass balance concepts, and include all known genes that contribute to metabolism, allowing for a full assessment of the metabolic status in a given organism.^59,60^ Computer modeling flux estimates, for example, have been demonstrated to improve the predictive capacity of ML in yeast and cyanobacteria whole-genome models.^61,62^ Similarly, genome-scale representations can be employed to recognize engineering objectives and focus on the realms of machine-learning algorithms.^63^ Another technique is to utilize machine learning to forecast the parameters employed in mechanistic models. Heckmann et al. demonstrated that enzyme turnover rates predicted by ML algorithms beat naively earmarked values at flux estimations.^64^ In one study, supervised ML algorithms and FBA were used in tandem to estimate bacterial central metabolism using input features from 37 different bacteria species, all of which had C13 metabolic flux data.^65^

One significant work attempted to comprehend the metabolism-regulatory mechanism by examining alterations in the metabolome and proteome of 97 kinase *Saccharomyces cerevisiae* mutants. The investigation demonstrated that in the absence of an underlying molecular framework machine learning can be employed to map alterations in regular enzyme expression profiles, which can subsequently be used to determine the metabolic phenotype.^66^ Burstein et al. used an ML and experimental strategy on the genome scale to find 40 new virulent bacterial effectors in *Legionella pneumophila*.^67^ Automation of significant aspects during fermentation is often unfeasible; however, soft sensors enable correlation between easily detected offline and online parameters to predict relevant offline variables in real time. One study employing structure additive regression (STAR) illustrates a model that can be created gradually, making it easier to analyze and adjust for operators.^68^ Furthermore, novel biosensor development strategies have been explored to build new soft sensors with potentially higher predictive ability over significant offline variables.^69,70^

Data are abundant in industrial bioengineering that is suitable for data mining and inclusion into ML models. Because of its capacity to extract the most significant predictors from vast, overlapping data sets, principal component analysis (PCA) has proven to be the most popular technique in the field.^71^ A significant amount of data in the industry and the literature needs to be normalized and standardized, which has shown to be a difficult challenge for biological systems data sets. For instance, Oyetunde et al. manually collected data containing 1200 cellular factories from approximately 100 papers to forecast the efficiency of an *E. coli*-based cell factory relying on all biologically significant parameters that were consistent among publications.^72^ They emphasized the need for standardization of data.

**Figure 5:**
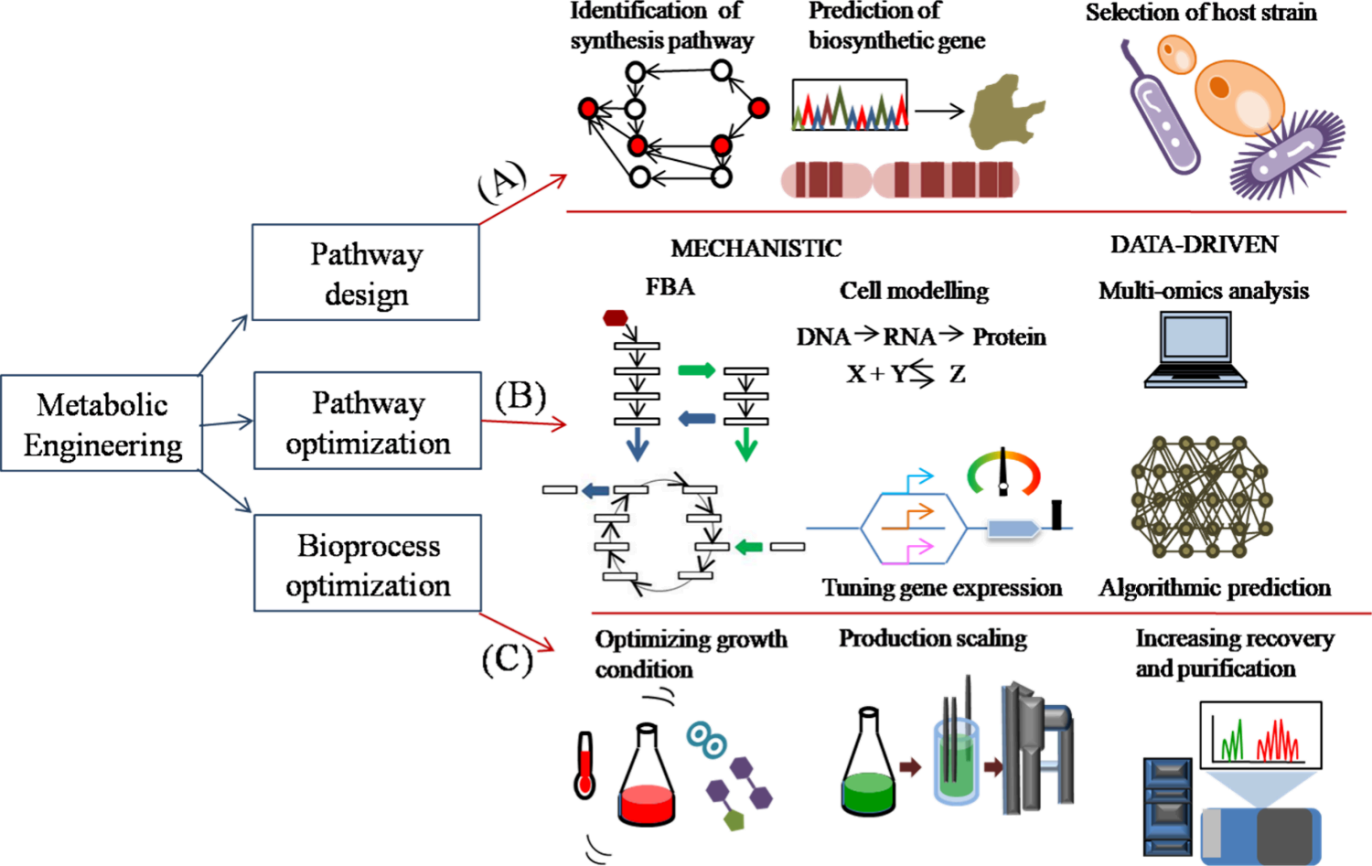
Applications of ML in metabolic engineering systems. In general, a metabolic engineering venture can be divided into three parts: (i) metabolic pathway design, (ii) boosting cells for production, and (iii) upgrading industrial operations for product yield. Numerous computing tools have been developed to direct designing throughout the process. (A) One can design pathways for the synthesis of target products by employing predicted genomic functions or proven chemical reactions. It can assist in locating hosts with inherent industrial applicability. (B) To increase production titer, frequency, and productivity, strains are engineered. Mechanistic techniques leverage the understanding of fundamental biology to predict metabolite synthesis. On the other hand, data-driven methods use patterns found in massive data sets to recommend improvements. Subsequent initiatives have attempted to integrate the two methodologies to boost predictive power. (C) The output of downstream bioprocesses is maximized. The time needed to adapt a lab strain for industrial output can be significantly decreased with in silico prediction.

One of the ultimate goals of metabolic engineering is to merge pathway design with host strain and culture condition optimization into a single pipeline (Figure 5). A standard workflow improves reproducibility, decreases the time required from project conception to realization,^73^ and allows for the usage of experimental automation to enhance throughput. Despite the benefits of a complete pipeline for metabolic engineering, there is a paucity of scientific literature explaining such methodologies. It opens the door for industries to establish unique techniques for engineering organisms for industrial purposes and for academics to investigate ways to use ML algorithms and techniques in streamlining the engineering of biosynthetic systems in organisms.

## Fundamental Building Blocks for DL Models

DL is a subset of ML that learns complicated patterns in data using networks with numerous layers of artificial neurons. An artificial neuron in ANNs is a mathematical function that simulates the activity of a biological neuron. ANN models are employed to classify data, recognize patterns, and accomplish multiple tasks. Although a single-layer neural network can be used for making predictions, extra hidden layers are used to enable optimization and increase accuracy. There are multiple DL architectures, and in this review article, I cover some popular ones employed in synthetic biology based applications.

### Multilayer Perceptrons (MLPs)

A standard ANN architecture employs a collection of “neurons”, and each neuron receives a series of numeric inputs. The inputs are multiplied by weight factors, and a constant termed bias is introduced. This value is subsequently processed by a nonlinear function to produce the neuron’s output. Initially, researchers utilized a sigmoid for the nonlinear function, but for computational performance, most recent DL network implementations employ ReLU (rectified linear units) for the neurons within the network’s hidden layers. There are typically multiple neurons, with the same inputs multiplied by various weights for each neuron. For instance, if the inputs are DNA sequence data, the weights regulate how each nucleotide influences the final output, including transcriptional activity. When given a multidimensional array as input, it can be unraveled into a vector (for example, a 4 × 50 matrix peeled into a 200-dimensional vector).

MLPs connect groups of neurons in fully linked networks so that the output of one layer enters the next. This hierarchical structure enables the detection of low-level traits in the early layers and far more complex characteristics in the later layers. The depth provided by numerous successive layers is where the prefix “deep” in the phrase “deep learning” derives from. Each neuron’s output is fully linked to all nodes on the next layer downstream in the MLP architecture. The network’s internal layers are referred to as hidden layers, while the final layer is known as the output layer. In contrast to the prior layers, which have several outputs, the output layer is unique in that it typically collapses to a single value or a limited number of values. In the network that delineates promoter data set to transcriptional activity, for example, the output may be a single integer that quantifies transcriptional activity.

### Convolutional Neural Networks (CNNs)

CNNs can save localized position data about how neighboring data is structured with one other. Furthermore, they employ a parameter-sharing approach in which the same model weights are used throughout the entire input. As a result, CNNs are particularly well suited to jobs like image processing, where neighboring pixels contain relevant information, and operations like edge detection must be executed effectively across the image. The input is convolved using a filter (or filters) and then fed through a nonlinear activating function for every convolutional layer of the network. Filters are valuable for detecting specific patterns.

Traditional filter-based analytic tasks use hand-selected numeric values in the filter to define features that a user believes are likely to be significant, such as edge detection. CNNs, on the other hand, employ filter parameters as model weights that the network learns (Table 1). CNNs often undertake sequential analysis actions that can abstract properties, including color gradients and patterns, using a set of convolution steps. Convolution layers are generally sandwiched between layers that conduct other mathematical functions, including pooling, which is employed to focus information by lowering data dimensionality. CNNs can also incorporate components of other network architectures, such as fully connected layers after convolutional layers.

**Table 1:**
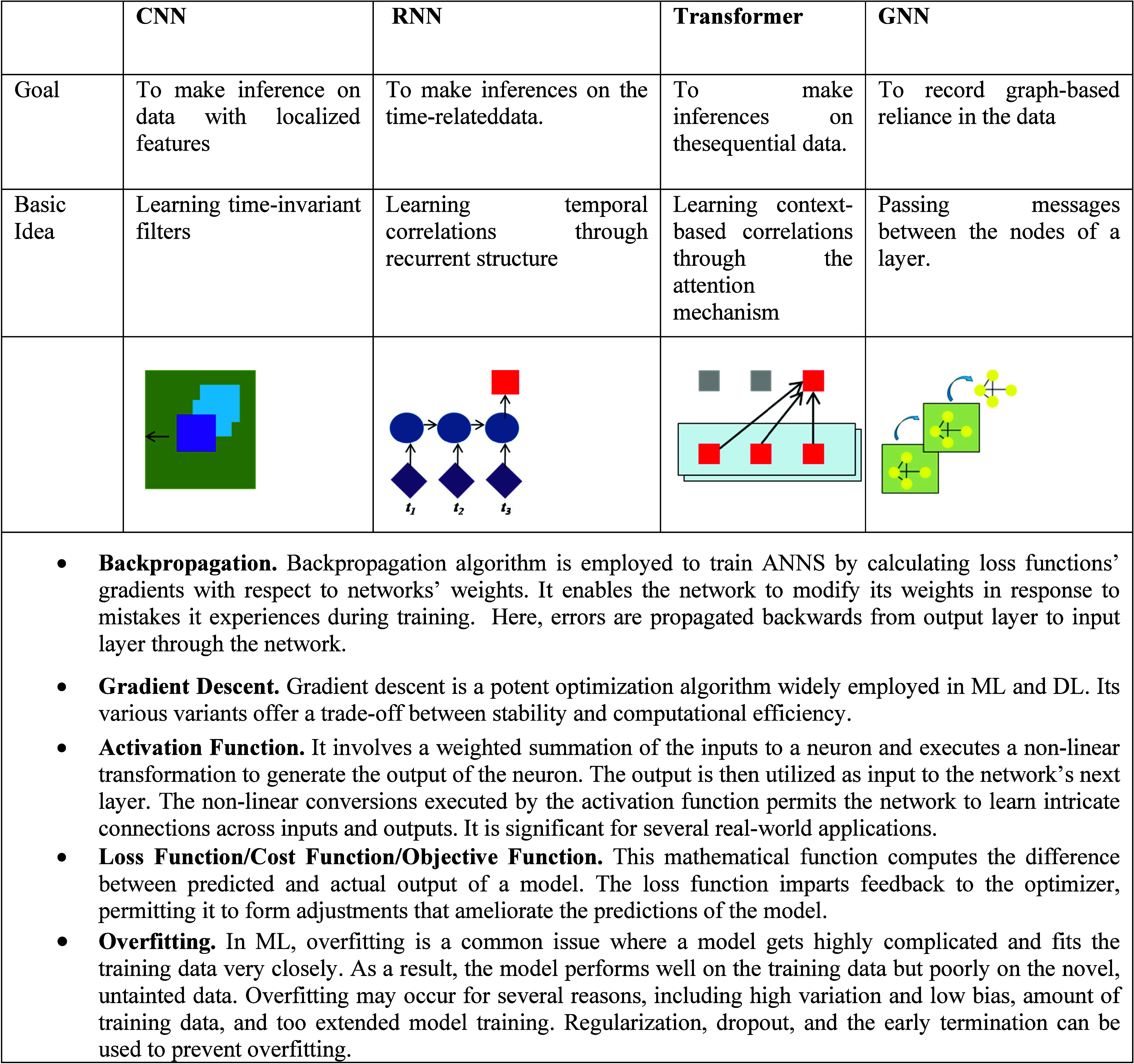
Some Key DL Architectures and Terms Used in the Manuscript

### Recurrent Neural Networks (RNNs)

RNNs are a type of model that is intended for usage with sequential data. They work by iterating through the data set and iteratively updating the model’s internal representation (or memory) based on the internal state’s content and the succeeding values in the input sequence (Table 1). These networks have traditionally been employed for language comprehension, where the organization of words is significant for context and interpretation. These networks are also suitable for analyzing biological time series information or sequence data. When processing DNA sequences, for example, the relative location of start and stop codons is crucial in determining protein expression. Nevertheless, the repetitive nature of these networks has significant drawbacks. Most crucially, because of the fading gradients issue, basic RNNs do not acquire long-term relations between elements that are located far apart in sequence space,^73^ and their iterative nature prevents parallelism in execution, restricting their scalability.

The introduction of LSTM (long short-term memory) networks significantly improved the performance of RNNs.^74^ LSTM models were created to improve RNNs’ limited temporal memory by including a long-term memory state in which the model must make clear-cut decisions regarding adding or removing information to the long-term memory. For instance, if a model is seeking to predict if a protein would be translated from a particular mRNA, the existence of a stop codon is likely to be stored in long-term memory until a downstream start codon is detected. More information on LSTM models is included in the review by Van Houdt et al.,^75^ and Angenent-Mari et al.^76^ provide an example of their use in synthetic biology.

### Transformers

The transformer is a more contemporary model built for sequential data that addresses the problems of limited memory experienced with RNN variants while also being computationally more methodical and parallelizable due to recurrence reduction. The transformer outperformed RNNs and LSTMs on all sequence-based tasks, demonstrating paradigm-shifting performance.^77^ Transformers have even outperformed CNNs on computer vision challenges,^78^ despite the fact that they were not initially designed for such tasks. This transforming performance is achieved by renouncing the notion of model memory and instead permitting the model to examine and produce outputs for every node in the whole sequence of data at the same time.

The model chooses which sections of the sequence to gather information from for each output. This is accomplished through a mechanism known as “attention”, in which the model may learn what information is relevant at each stage in the sequence and concentrate on passing that knowledge forward (Table 1). A model anticipating the behavior of a short RNA that may form secondary structures, for example, is likely to focus on sequences that are supporting to the sequence of relevance (e.g., outputs for “CGA” will contain a significant amount of data from the other section of the sequence having “UCG”). The mathematical intricacies of the attention mechanism are outside the scope of this review article, but readers should read Chaudhari et al.^79^ for further information.

### Graph Neural Networks (GNNs) and Geometric Approaches

Learning methods for image and sequence data take advantage of the data’s methodical Euclidean structure and the intuitive notion of spatial locality that it provides. These structural attributes are not shared by other structured data, including secondary structure graphs of RNA and DNA, structural formula graphs of molecules, and atomic coordinate data for proteins. Nonetheless, they possess their own symmetries and notions of locality that can lead to developing learning frameworks. GNNs can expand the sharing of information in Euclidean neural networks to the graph structure, offering a scaled and generalized method for conveying information between nodes via the irregular edge connections that operate to encode the locality of the structure (Table 1).

It enables the learning of high-quality renderings of a structured data that can then be employed for edge prediction tasks or node labeling or pooled across the structure and supplied into an MLP to conduct regression or classification at the molecular scale. Bronstein et al.^80^ provide a thorough and inclusive primer for understanding ML from a geometric standpoint, and Zhou et al.^81^ provide a description of the intricacies of GNN formation.

### Generative Models

Generative models^82−84^ are a class of artificial intelligence models that aim to learn and replicate patterns present in the data they were trained on. These models are trained on a data set and then used to generate new, similar data. There are various types of generative models, and they operate in different ways. Some common types include the following.

### Generative Adversarial Networks (GANs)

GANs consist of two neural networks, a generator, and a discriminator, which are trained simultaneously through adversarial training. The generator creates synthetic data, and the discriminator’s role is to distinguish between real and generated data. The competition between these two networks helps the model generate increasingly realistic data.^85^

### Variational Autoencoders (VAEs)

VAEs are probabilistic generative models that learn a probabilistic mapping between the data space and a latent space.^86^ They aim to encode input data into a probabilistic distribution in the latent space, allowing for the generation of new samples by sampling from this distribution.

### Autoencoders

Autoencoders consist of an encoder and a decoder. The input data is compressed by the encoder into a latent space representation, which the decoder then uses to recreate the original data. While not inherently generative, variations like variational autoencoders can be used for generative purposes.

### Boltzmann Machines

Boltzmann machines are a type of stochastic recurrent neural network. They use a network of binary-valued nodes and learn to model the probability distribution of the training data.^87^ They can be used for generating new samples.

Generative models have various applications, such as image and text generation, data augmentation, style transfer, and more. They play a crucial role in unsupervised learning tasks and can be used to explore and understand the underlying structure of the data they are trained on.

## Applications of DL in Synthetic Biology

In this section, I investigate examples of deep learning in synthetic biology research (Figure 7A). I discuss current advances in the design of biological parts, imaging applications, structure-based learning, optimal experimental design, and implementations of biomolecular neural networks.

### Design and Simulation of Biological Components

Deep learning has recently made substantial progress in predicting the function of biological components, like ribosome binding sites (RBSs), promoters, and 3′ and 5′ untranslated regions (UTRs).^4,76,88−95^ Since these components are frequently constrained in length, for instance, approximately 50 nucleotides for a 5′ UTR sequence or approximately three hundred for a promoter-DNA synthesis can be used to create massive randomized or semirandomized libraries whose function can be assessed using massively parallelized reporter assays combined with the next-generation array. The capacity to synthesize enormous libraries is an excellent example of how synthetic biology methods may produce training sets for data-hungry models.

Deep learning algorithms have recently been utilized to detect^96,97^ and potentially interpret protein sequences^98^ in genomes from superior-quality experimental data sets. DeepRibo, a deep neural network (DNN)-based technique that uses increased ribosome profiling coverage indicators and potential open-reading frame patterns to map and detect translated open-reading frames in the prokaryotes is one approach currently being used to locate protein sequences. REPARATION, a similar tool, uses a random forest classifier to do the same task.^99^ After discovering new proteins, functional interpretation of their sequences can be accomplished using DNN-based techniques such as DeepEC, which uses a protein sequence to determine enzyme commission numbers (EC numbers) quickly and precisely.^98^ EC numbers categorize enzymes according to the chemical reactions they catalyze and assist in studying enzyme functions. Alternative EC number prediction algorithms, in addition to DeepEC, are Cat Fam,^100^ DEEPre,^101^ ECPred,^102^ DETECT v2,^103^ PRIAM,^104^ and EFI CAz2.5.^105^

**Figure 6:**
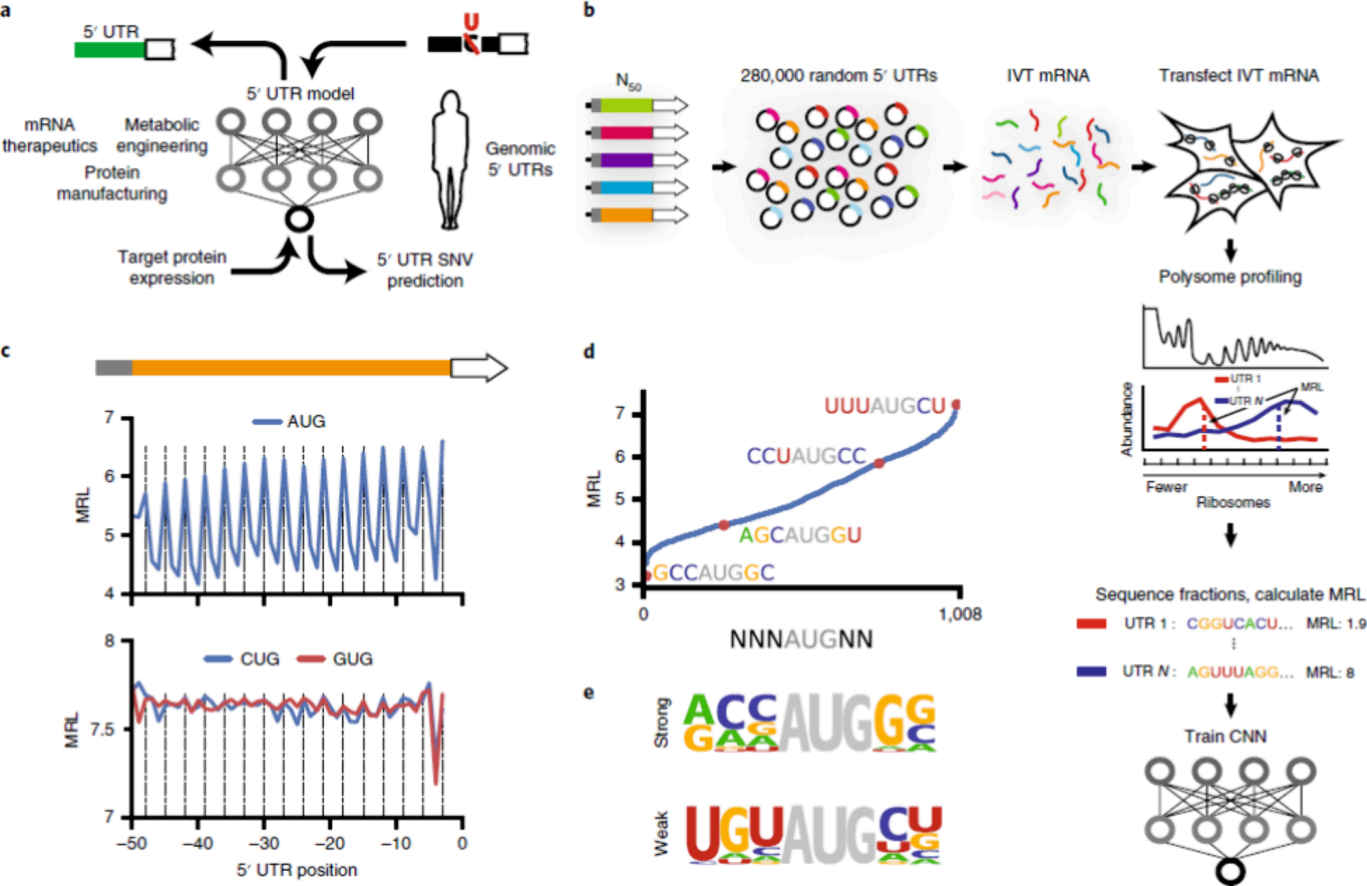
Library consists of 280,000 random 50 nucleotide oligomers as 5′ untranslated regions (UTRs) for enhanced green fluorescent protein (eGFP). (A) Shows the usage of a 5′ UTR to assess the potential of 5′ UTR single nucleotide variants (SNVs) and engineer state-of-the-art sequences for prime protein expression. (B) The construction of the library of 280,000 members by the insertion of a T7 promoter accompanied by 25 nucleotides of stipulated 5′ UTR pattern, a random 50-nucleotide pattern, and the eGFP coding sequences (CDSs) into the backbone of a plasmid. In vitro transcribed (IVT) library mRNA was generated by in vitro transcription from a linear DNA template acquired by a polymerase chain reaction from the plasmid library. HEK293T cells were transfected with IVT library mRNA; cells were collected after 12 h; and polysome fractions were then collected and sequenced. In vitro transcribed library mRNA transfected HEK293T cells were recovered after 12 h, and then polysome profiling was conducted. For each UTR, read counts per fraction were utilized to calculate mean ribosome load (MRL), and the resulting information was employed to train a CNN. (C) The uAUGs (out-of-frame upstream start codons) decrease ribosome loading (positions that are in frame with the enhanced green fluorescent protein coding sequences are shown by the vertical lines). Analogous but very weak periodicity was observed in the case of GUGs and CUGs. (D) Shows the repressive efficacy of all out-of-frame variance of NNNAUGNN. (E) Shows the nucleotide frequencies deliberated for the 20 least repressive (weak) and most repressive (strong) translation initiation site sequences. Adapted with permission from ref (90). Copyright 2019, Nature Publishing Group.

Sample et al.^90^ created Optimus 5-Prime, a DL model that precisely predicts how the 5′ UTR sequence regulates ribosome loading (Figure 6). Even though data sets relating sequence to translation performance from endogenous human 5′ UTRs exist,^106,107^ these innate data sets are not best suited for model training since sequences with detrimental effects are plausible to be underrepresented in innate illustrations, and endogenous transcript data are not diverse enough to capture a wide range of expression profiles. To address these concerns, Sample et al. synthesized and evaluated data from a 280,000-member library of random 50-nucleotide 5′ UTR segments upstream of the green fluorescent protein coding region (Figure 6). The Optimus 5-Prime model was trained using data from transfected HEK293T cells, with inputs being one-hot encoding renditions of the 5′ UTR sequencing and the output being the average ribosome load values. The researchers utilized CNN, and the model performed admirably, predicting up to 93% of the test set’s average ribosome loading values.

For promoter designs, similar strategies that integrate DNA synthesis, DL, and massively parallel reporter assays have been applied. Traditionally, synthetic biologists have used a restricted number of native regulators in their construction designs. Although there are artificial promoter libraries,^108−110^ they are typically variants of existing sequences, like those obtained through mutagenesis, limiting diversity. Moreover, because they are underrepresented in natural situations, there is a scarcity of strong promoters. Kotopka and Smolke^4^ used massively parallel reporter tests to characterize a promoter variant library. The design kept the conserved sequences within the promoter and randomly generated the rest (∼80% of the sequences).

It demonstrates a potential method for accessing bigger sequence spaces by combining sensible and randomized designs. The researchers utilized a blend of high-throughput DNA sequencing (FACS-seq) and fluorescence-activated cell counting to categorize cells based on their expression levels, then sequenced the promoter regions within every bin. These data were utilized to train a CNN, which takes a DNA sequence as input and predicts activity. Generally, the model predictions translated well to test data, with R2 values greater than 0.79 for all libraries, a noteworthy achievement given the complexity of the sequences. This method of employing massively parallel reporter assays is broadly applicable. Jores et al.^111^ created synthetic promoters for plant species such as *Arabidopsis*, sorghum, and maize, and instructed a CNN to forecast promoter strength. MPRA (Massively parallel reporter assays) are not the only technique to create big data sets, and alternative ways may be less prone to processing biases. Hollerer et al. employed genetic reporters to generate a large data set that correlates directly sequence to function, which they then used to design a deep learning model that accurately predicts the translation pursuit of an RBS.^91^ The researchers constructed a library of 300,000 bacterial RBSs and inserted them upstream of a site-specific recombinase, which flips a specific DNA sequence in a region close to the recombinase.

The researchers were able to test function by assessing the proportion of constructs that had undergone recombination for each RBS variant by sequencing the area comprising both the RBS and the recombinase domains. This data set was utilized to instruct a ResNet53 (a CNN version), which resulted in a model that prognosticated the RBS function with inflated accuracy (R2 = 0.927). It is worth mentioning that the basic approach utilized to construct a physical DNA-recorded linkage between DNA sequence and gene regulatory element functionality is not limited to RBS optimization but could also be used for translational or transcriptional biosensor design or promoter sequence optimization. Despite the high promise of employing synthetic sequences to produce diverse libraries, this strategy has certain limitations. Deep learning studies have repeatedly encountered the difficulty that employing purely randomized sequences sequels a large number of sections that do not work. On the other hand, because natural elements are biased in their depiction, exclusively random parts are likewise prone to fail. Researchers have worked around this issue by adopting semirational strategies, including interspersing regulatory elements believed to give functional regulators with randomized sequences^2^ and then employing model predictions to choose libraries augmented for elements with an intermediary or strong activity.^110,91^ Furthermore, the sequence length will eventually limit the library’s diversity. The capability to synthesize and sequence larger sections may sequel reduced coverage and biased data quality in the case of lengthier sequences. Furthermore, researchers must negotiate between sequencing read length, sequencing depth, and library size.

The advantages of emphasizing particular sequence areas as “modules” must be balanced against the reality that gene regulation is complicated. Zrimec et al.^112^ demonstrated the importance of interactions between coding and noncoding domains in ascertaining gene expression levels. However, they illustrated that DNA sequences can be utilized to assess mRNA abundance straight with some precision (R2 = 0.6 on the mean across a wide range of model organisms, such as *Saccharomyces cerevisiae*, *Arabidopsis thaliana*, *Homo sapiens*, and others), the interplay between regulatory motifs, rather than the motifs themselves, ascertained mRNA abundance. These findings serve as a straightforward reminder that biological components do not function in isolation.

### Generative Strategies for Novel Synthetic Components

**Figure 7:**
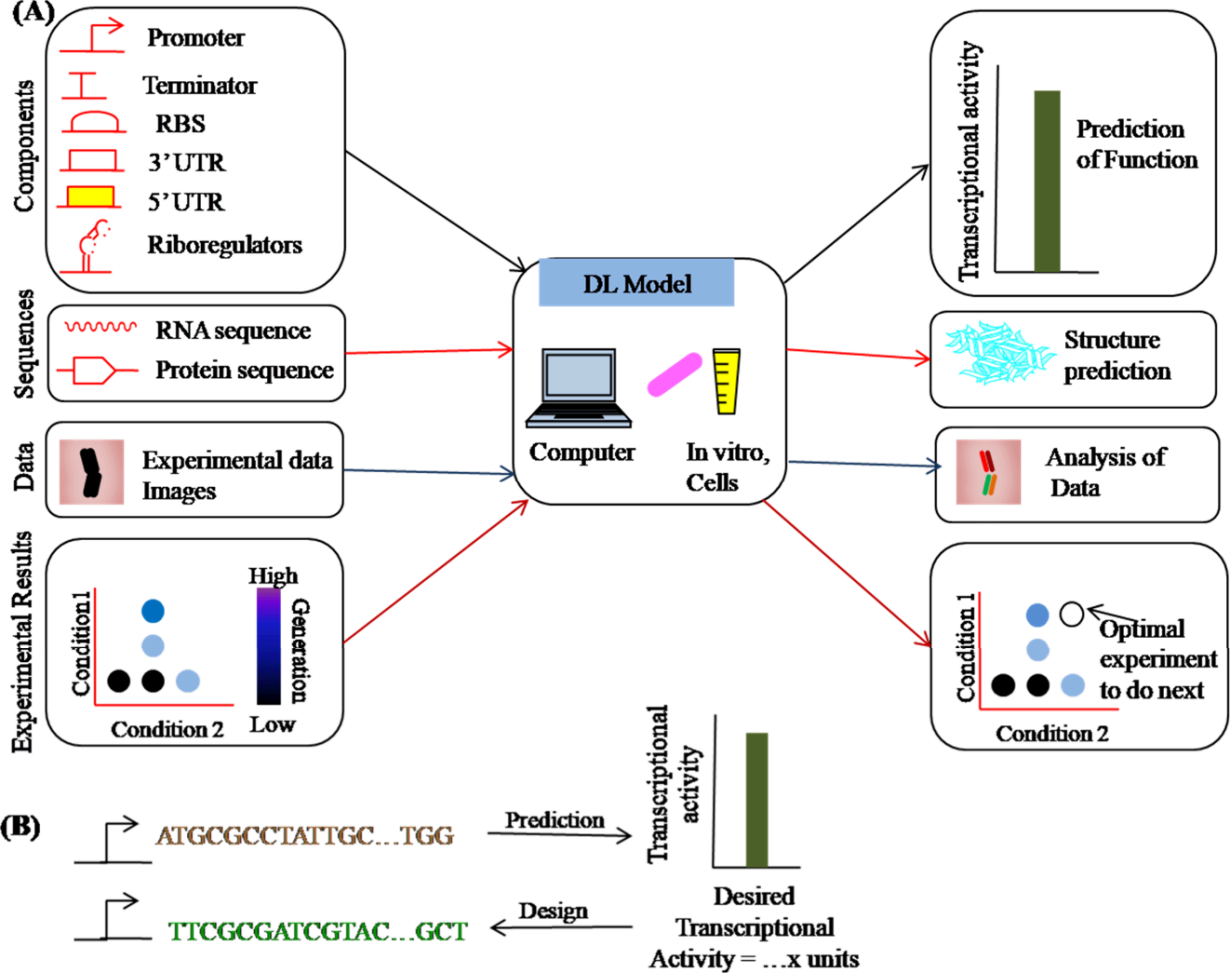
DL enabled applications of synthetic biology. (A) Representative cases of pertinent inputs to DL networks and their allied output predictions. (B) Given a fresh input, predictions can be made using deep learning. Using a desired output as a starting point, models can likewise be utilized in reverse to produce new designs.

Synthetic biology applications are typical prerequisites for a model to be predictive as well as generative (Figure 7B). Nondeep learning applications have been highly beneficial to the engineering biology field. The RBS calculator,^113^ for example, may produce unique designs based on a thermodynamic framework, and synthetic 5′ UTR sequences have been auspiciously generated using genetic algorithms.^90^ Mechanistic modeling techniques are very potent; nevertheless, they require the professional expertise of which attributes contribute to performance. Deep-learning-based generative techniques are an attractive field of research, as these tools approach the capacity to work backward, for example, from translation efficiency specifications to candidate sequence designs. Kotopka and Smolke^2^ employed a CNN model to execute sequence-design approaches in their research on yeast promoters, demonstrating that the best algorithms provided potent synthetic constitutive and inducible promoters.

Traditional techniques to design optimization, on the other hand, might be vulnerable to practical drawbacks such as computing inefficiency and a proclivity to become stuck at classical optimization minima. Moreover, these algorithms have no limitations on sequencing diversity, which might be troublesome for generating a large number of distinct library variants. Deep generative models, which include models such as variational autoencoders, generative adversarial networks, and autoregressive models, have the capability to fill these gaps. Linder et al.^114^ built a deep exploration network framework as an example of this method. They used a similarity metric that discourages sequence similarities that surpass a threshold to maximize fitness for the intended function while simultaneously explicitly emphasizing sequence diversity. Generative models have also shown success in the field of peptide engineering for simple challenges involving short-chain peptides, such as antibacterial peptide design.^115,116^

### Applications Based on Structure

Rapid advancements in the field of geometric DL have facilitated a surge in exploration into structure-to-function learning in the field of biotechnology. The AlphaFold2 protein structure predicting model,^117^ which promises protein structure prediction fidelity high enough to be used as a successor for costly and time-taking protein crystallography, is perhaps the most high-profile example. As inputs, the model uses the protein sequence and several sequence alignments akin to proteins to learn about three separate data structures: (i) a sequence-level representation, (ii) a pairwise nucleotide interaction representation, and (iii) the protein’s atom-level three-dimensional (3-D) structure production. The 3-D structure is depicted as a cloud of unconnected nodes that correspond to the backbone constituents of each nucleotide and their respective amino acid side chains. To make use of the translational and rotational symmetries inherent in 3-D space geometry, a geometric equivariant attention mechanism is applied. Protein sequence-function mapping and engineering are further aspects of interest in the protein arena.^118−122^ Gelman et al.^11^ reported that on receiving training on data from deep mutational scanning tests, deep networks, including convolutional networks, can effectively predict function for new unidentified sequence variants.

When compared to the protein folding problem, the lack of known structural data makes predicting the 3-D RNA structure more difficult. Although over 100,000 protein structures have been identified, only a few RNA structures have high-fidelity structures. Townshend et al.^123^ used an intriguing strategy to overcome this restriction, in which they reframed the task as one of scoring the structural predictions given by the FARFAR2 algorithm rather than predicting the structure of RNA end-to-end with a DL model. It allowed for a substantial augmentation of the available data set, which only contains 18 RNA structures. It is insignificant to build thousands of proposed structures for every RNA molecule in the training data set, instead of learning to identify the similarity between proposed structures and the rational truth. The learned structural scoring function, termed the Atomic Rotationally Equivariant Scorer (ARES), outperforms existing nonmachine learning procedures in terms of accuracy. In recent years, structural modeling on small-molecule graphs has grown fast in the realms of drug discovery^124,125^ and drug repurposing.^126^ Stokes et al.,^127^ for example, used graph neural networks (GNNs) in tandem with screening assays to predict antibiotic activity in small molecules, identifying a new medication termed halicin as an efficient antibiotic in animal models.

Protein engineering entails either synthesizing new proteins or altering the sequence and structure of existing proteins.^128^ Large DL models are splendidly capable of learning various properties of proteins.^128,129^ Better wild-type templates can be generated by employing structural data. The usage of a local structural environment for identifying sites suitable to optimize wild-type proteins is one promising approach for this purpose. Recent research based on plastic degrading enzymes showed the power of this strategy.^130^ For determining which sites, the estimated probabilities of wild-type AÃs (amino acids) were relatively low, and Lu et al.^130^ employed the MutCompute^131^ algorithm. This suggests that certain alternative AÃs may be more “suited” to the appropriate structural microenvironment. Dauparas et al. trained ProteinMPNN (a graph based NN) on 19,700 high resolution single chain structures from PDB. They demonstrated that ProteinMPNN can extricate different failed designs by advising optimized protein sequences for the given templates.^132^ In a recent study, SoluProt^133^ and the enzyme miner integrated pipeline were employed for mining industrially pertinent haloalkane dehalogenases^134^ and fluorinases.^135^

### Applications for Imaging and Computer Vision

DL has enabled unprecedented development in computer vision.^136^ Imaging applications in synthetic biology can involve automated detection of appropriate ties within an image, including colony growth on a plate or microscopy data analysis. Classification (for example, determination of the existence of a colony) and segmentation (for example, identifying the sets of pixels related to each cell in an image) are two examples of image analysis tasks. Classification is the simplest of these tasks, and basic CNN algorithms from computer vision, such as AlexNet,^137^ LeNet-5,^138^ and ResNets,^139^ were developed for it. Deep neural networks with numerous parameters (for example, AlexNet makes use of approximately 60 million parameters) were usually used in these classical algorithms. To decrease this complexity, smaller versions, including MobileNetv2^140^ (approximately 3 million parameters), have been developed, providing a realistic alternative.

Locating the exact position of an entity within an image is a more complicated task that is especially useful for quantification. Segmentation, for example, can be used to locate the position of cells within microscope images so that fluorescence measurements can be retrieved. With the advent of the U-Net algorithm,^141^ a CNN that performed extraordinarily well on biological data, the field witnessed a big advance. DeepCell,^142^ YeaZ,^143^ DeLTA,^144,145^ CellPose,^146^ and MiSiC^147^ are some significant DL algorithms that are applicable for single-cell resolution data.^146^ Image analysis algorithms can also handle more powerful analytics tasks, including monitoring cells from frame to frame in time-lapse photos and dealing with 3D image data.

### Optimal Experimental Design

When compared to other domains, data tagging for synthetic biology challenges is frequently quite expensive, requiring professional knowledge of the subject and, in some cases, sophisticated laboratory-based data-gathering systems. This cost is especially problematic for deep learning models requiring outstanding training data. It increases interest in ensuring practitioners do not squander time and resources in classifying data, not adding much to a model. The selection of appropriate data to label or tests to run is an optimum experimental design termed active learning in the ML community. The usage of this method to solve DL problems can greatly minimize data set development costs.^148,149^

**Figure 8:**
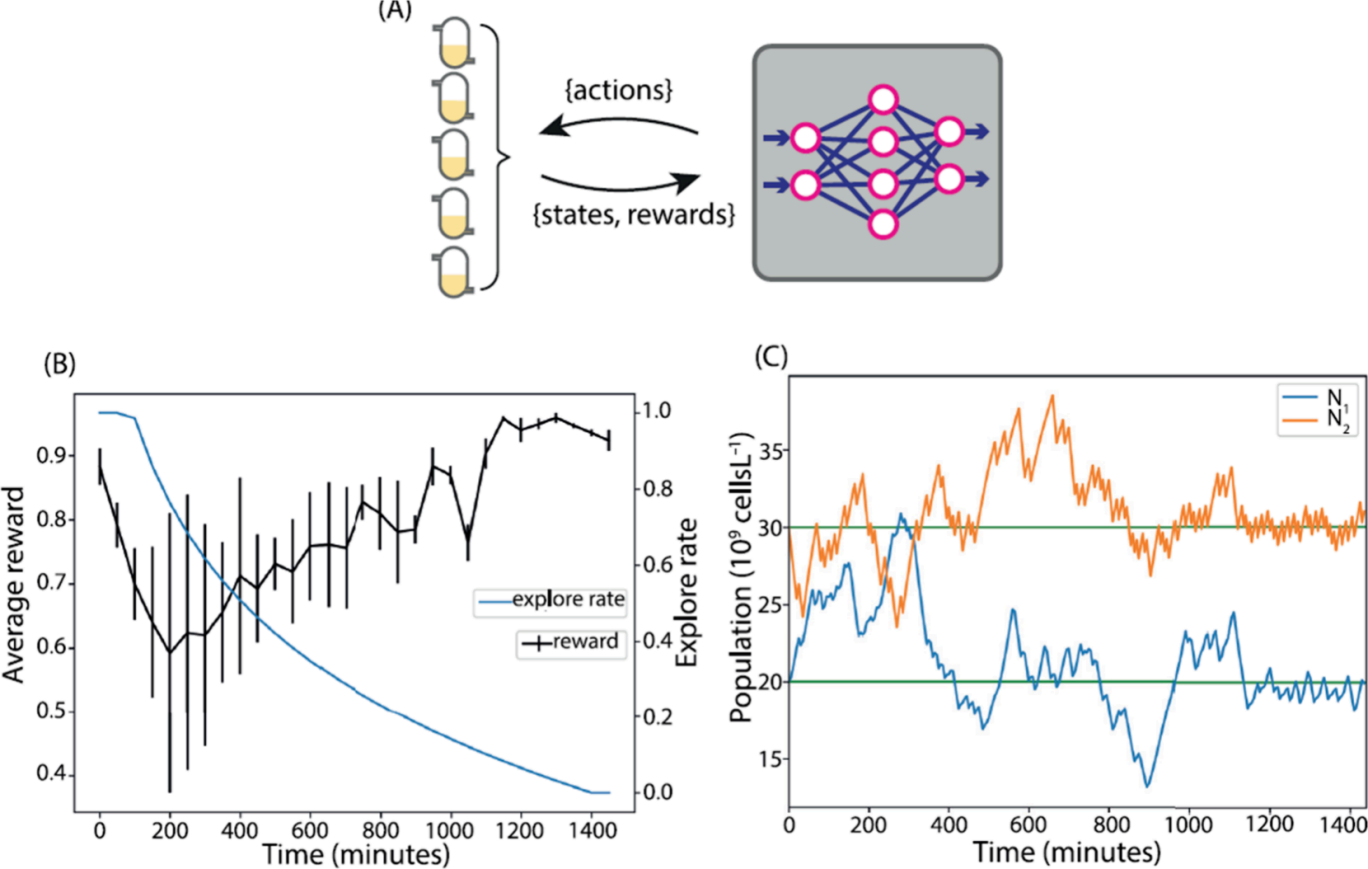
Learning a proposed plan in 24 h. (A) Training of reinforcement learning agent was conducted online for 24 h on a model comprising five parallel chemostats. (B) Shows the reward obtained from the surroundings. Despite a little standard difference in reward, all five chemostats had been relocated to the intended population levels by the completion of the simulation. (C) Exhibit the population curve of one chemostat. The population levels change, and random actions are conducted throughout the exploration phase. When the exploring rate declines, the population levels approach the target values. Reproduced from ref (150) (an open access article distributed under the terms of the Creative Commons Attribution License). Copyright 2020, Treloar et al.

DL algorithms for optimal experimental design are not yet extensively employed in engineering biology; nonetheless, the ability of laboratory automation and initial findings based on simulation indicates that this is a viable area for future research. Treloar et al.^150^ employed deep reinforcement learning for controlling a simulated chemostat representation of a microbial coculture developing in a continuous bioreactor. The authors showed that by running five bioreactors in tandem for 24 h a reasonable control policy can be gained and that deep reinforcement learning can be employed to determine the best pattern of inputs and control actions to pertain to a continuous chemostat to increase the product performance of a microbial coculture bioprocess. It is a computational example of a DL-driven optimal experimental design in which reinforcement learning is employed to estimate near-optimal patterns of bioreactor inputs to manage a complicated system (Figure 8). Future work in optimum experimental design can rely on existing ML algorithms, such as those used in metabolic engineering applications.^54,63,151−153^

### Biomolecular Applications of DL Networks

Although DL models are generally executed using computers, new research has shown that ANN mimics can be built utilizing biomolecular elements. These designs create biochemical systems and live cells that can compute and “learn” to resolve simple benchmark optimization issues. One of the primary reasons for this is that inducible gene expressions to chemical inducers often resemble a sigmoidal function of the inducer concentration and can therefore act as the nonlinear function in the neuron model.

On this basis, Moorman et al.^154^ introduced the theoretical design of a biomolecular neural network which is a dynamical chemical reaction network that reliably executes ANN computations and illustrated its applicability for classification tasks. The authors emphasized the significance of molecular entrapment in attaining negative weight values and the sigmoidal activation function in its elementary unit known as a biomolecular perceptron. Samaniego et al.^154,155^ theoretically showed that interlinked phosphorylation/dephosphorylation cycles can function as multilayer biomolecular neural systems. From an application point of view, they created signaling networks that potentially function as linear and nonlinear classifiers.

**Figure 9:**
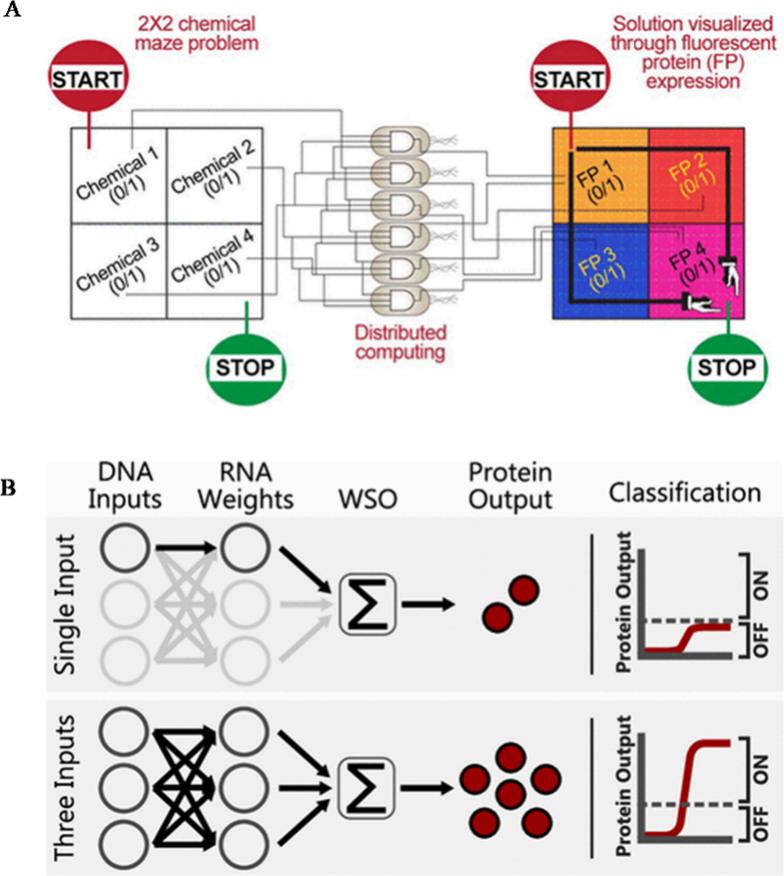
(A) Application of the distribution of simple genetic circuits among bacterial populations to solve chemically produced 2 × 2 maze issues by selectively articulating four distinct fluorescent proteins. Reproduced with permission from ref (158). Copyright 2021, American Chemical Society (https://pubs.acs.org/doi/10.1021/acssynbio.1c00279, further permissions related to the material excerpted should be directed to the ACS). (B) Synthetic in vitro TxTl-based perceptron comprised of WSO linked to a thresholding function. Reproduced with permission from ref (159). Copyright 2022, American Chemical Society (https://pubs.acs.org/doi/10.1021/acssynbio.1c00596, further permissions related to the material excerpted should be directed to the ACS).

Sarkar et al.^156^ experimentally applied a single-layer ANN in *Escherichia coli* (*E. coli*) cells. They demonstrated the application of engineered bacteria as ANN-empowered wetware capable of performing complex computing operations, including multiplexing, demultiplexing, majority functions, encoding, decoding, and Feynman and Fredkin gates. In another study, Li et al.^157^ applied ANNs to a consortia of bacteria interacting via quorum-sensing molecules. They employed these engineered bacteria to identify 3 × 3 binary patterns. Sarkar et al.^158^ used elementary genetic circuits dispersed across different bacteria to solve chemically derived 2 × 2 maze issues by selectively articulating four distinct fluorescent proteins, illustrating the feasibility of using engineered bacteria to conduct distributed cellular computing and optimizations (Figure 9A). van der Linden et al.^159^ used genetic engineering to create a perceptron competent of binary classification. It was accomplished by constructing a synthetic in vitro transcription and translation (TxTl)-based weighted sum operation (WSO) circuit linked to a thresholding function employing toehold switch riboregulators. The synthetic genetic circuit was employed for binary classification, which involves expressing a single output protein only if the necessary minimum of inputs is exceeded (Figure 9B).

Pandi et al.^160^ described a method for biological computing using metabolic components applied in whole-cell and cell-free systems. The implementation depends on metabolic transducers, which are analog adders that perform a linear combination of the concentrations of numerous input metabolites with customizable weights and are used to generate metabolic perceptrons. Relying on this, the authors constructed two four-input metabolic perceptrons for binary classifying metabolite combinations, providing the framework for quick and scalable multiplex sensing using metabolic perceptron networks. Faure et al.^161^ recently demonstrated that artificial metabolic networks may be utilized to create RNNs that can be trained to anticipate growth rates or an organism’s consensual metabolic behavior in response to its surroundings. Because the proposed artificial metabolic networks can improve multiple objective functions, they might be employed to find optimal solutions in a variety of industrial applications, including finding the best media for the bioproduction of desired compounds or engineering microorganism-based judgment devices for multiplexed identification of metabolic biomarkers or environmental contaminants. Such biological evidence of ANNs and ML paradigms executed at the biomolecular level opens routes for novel research into the engineering of living cells for resolving complex computing, governing, and optimization problems.

## Challenges

AI has started to find its way into many synthetic biology applications, but significant sociological and technological barriers remain between the two sectors. Large volumes of high-quality data are needed for machine learning to train algorithms. Getting these data is the major challenge in synthetic biology. Large-scale data generation is a serious difficulty in synthetic biology sectors where deep learning models are known to be notoriously data hungry. Training data, imbalanced data, uncertainty scaling, catastrophic scaling, overfitting, and vanishing gradient problem are some of the issues^162−164^ of DL.

### Technological Challenges

**Figure 10:**
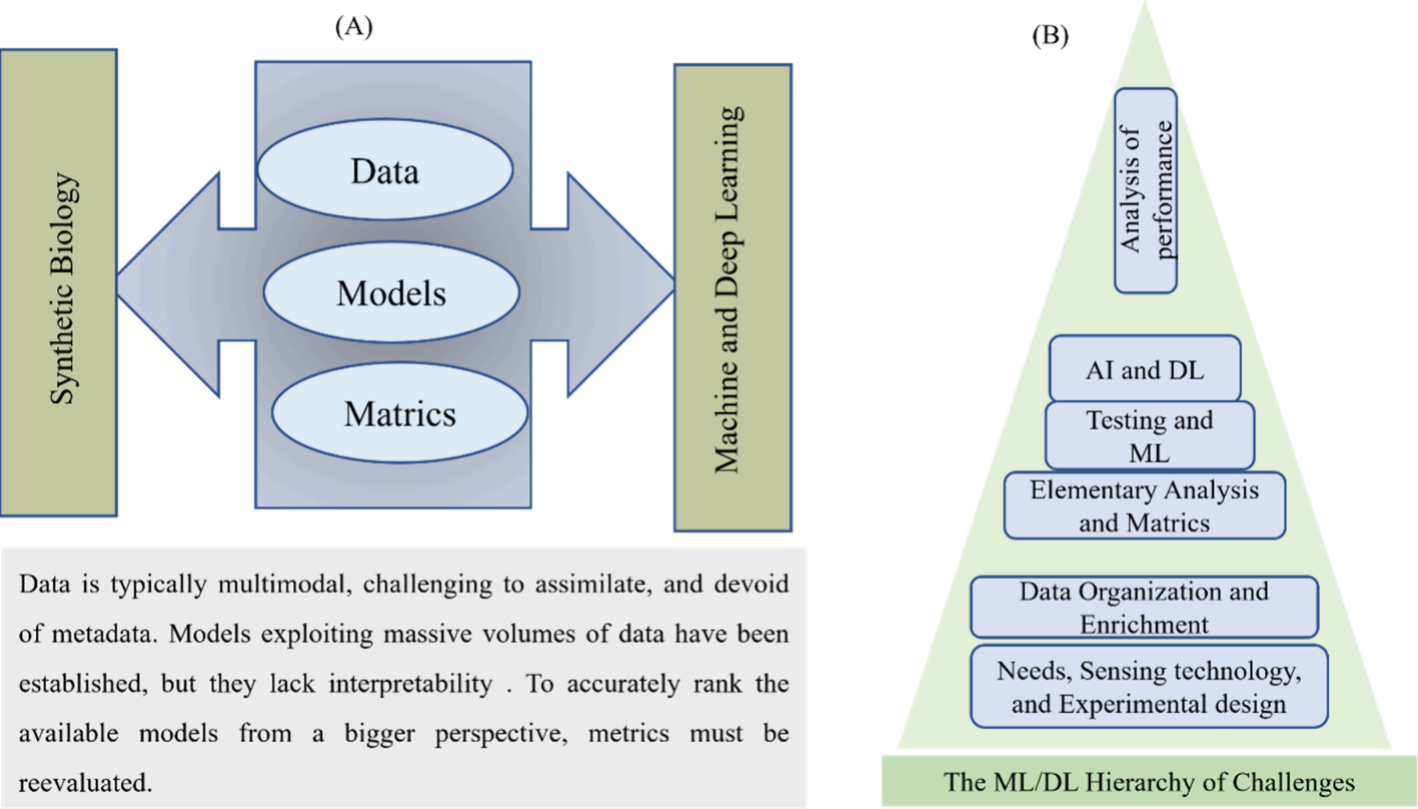
(A) Challenges of amalgamating ML/DL techniques with applications of synthetic biology. (B) A standard ML/DL framework can help synthetic biology research. The intermediate stages are typically the center of attention, yet the foundation is critical and requires massive resource investment.

The technical hurdles of applying AI to synthetic biology (Figure 10A) are as follows: (i) data is dispersed across multiple modalities, hard to combine, nonstructured, and generally lacks the scope in which it was gathered; (ii) models likely require more data than is typically gathered in a single trial and inadequate predictability and turmoil quantification; and (iii) there are no measurements or benchmarks to accurately assess prediction accuracy in the higher range task to be performed. Moreover, investigations are typically planned to investigate only positive outcomes, confounding or biasing the model’s judgment.

#### Data Challenges

The first big obstacle to combining AI and synthetic biology is the lack of adequate data sets. To use AI for synthetic biology, massive amounts of classified, organized, high-quality, and context-rich data from investigations are required. Despite advancements in establishing databases,^165^ including varied biological sequences (like whole genomes) and characteristics, there remains a dearth of labeled data. I refer to “labeled data” as phenotypic data that has been mapped to assessments that capture its bioactivity or cellular responses. The inclusion of such metrics and labels, as in other sectors, will accelerate the maturation of AI/ML and synthetic biology solutions to surpass human competency. The issue of irreproducibility in scientific research is indeed a serious concern that has garnered increasing attention in recent years. Irreproducibility refers to the inability of other researchers to replicate the results and findings of a study using the same methods and data. This problem undermines the reliability and credibility of scientific research, as reproducibility is a fundamental principle of the scientific method. Numerous research reports claim a significant outcome; however, their results cannot be reproduced. Studies showing that research is frequently not repeatable have drawn more attention to this issue in recent years. For instance, a 2016 Nature survey^166^ found that over 70% of scientists in the field of biology alone were unable to replicate the results of other scientists, and almost 60% of researchers were unable to replicate their own findings. Addressing irreproducibility requires a collaborative effort from researchers, institutions, journals, and funding agencies to establish a culture of transparency, rigor, and accountability in scientific research.

A lack of funding in data engineering is partly to blame for the scarcity of suitable data sets. Artificial intelligence advancements typically eclipse the computing infrastructure needs that underpin and ensure its success. Data engineering is a prime component of the basic infrastructure often regarded as the pyramid of needs^167^ (Figure 10B) by the AI community. Data engineering includes the phases of experimental design, data gathering, organization, accessing, and interpretation. Most AI application examples include a consistent, systematic, reproducible data engineering process. While we can currently collect biological data on an unprecedented scale and in unprecedented detail,^168^ this data is not always instantly suited for machine learning. Many barriers remain in the way of the acceptance of society standards for storing and sharing measurements, experimental procedures, as well as other metadata that would render them more accessible to AI approaches.^165,169^ To make such norms quickly deployable and to encourage shared metrics of data performance analysis, intensive formalization work and agreement are required. In brief, AI models necessitate reliable and comparable measurements throughout all trials, which lengthens the experimental timeline. This prerequisite adds a tremendous burden to experimentalists, following intricating protocols to produce scientific breakthroughs. As a result, the long-term demands of data collection are sometimes sacrificed to achieve the short timelines that are frequently placed on such initiatives.

It frequently leads to sparse data sets that reflect only a portion of the various layers that comprise the omics data stack. Data representation has an increasing impact on the capacity to merge these siloed sources for modeling in these circumstances. Today, tremendous effort is expended across a wide range of industry verticals to gather and organize unmanageable digital data for analysis through data cleansing, data set alignment, extraction, transformation, and load operations (ETL). These tasks consume nearly half to 80% of a data scientist’s time, reducing their potential to extract insights.^170^ Coping with a wide range of data forms (data multimodality) is problematic for researchers of synthetic biology, and the intricacy of pretreatment tasks increases considerably as data variety increases compared to data volume.

#### Algorithmic/Modeling-Based Challenges

Several efficient models driving current AI developments (for example, in natural language processing and computer vision) are not flavorful when examining omics data. When used for data obtained in a given experiment, common approaches of these models can undergo the “curse of dimensionality”. For instance, a single researcher can generate proteomics, transcriptomics, and genome data for an entity under a specific circumstance, yielding over 12,000 observations (dimensions). For such a study, the number of annotated events (e.g., failure or success) typically ranges from tens to hundreds. For such wide data types, the system dynamics (time resolution) are rarely recorded. These measuring gaps make drawing conclusions about complicated and dynamic systems difficult.

Although omics data has similarities and contrasts with other data types, including text data, sequential data, and network-based data, traditional approaches are not always relevant. Positional encoding, constraints, and complicated interaction patterns are examples of shared data properties. However, there are significant distinctions, including basic representation, the context needed for relevant analyses, and the accompanying normalizations among modalities to create biologically meaningful comparisons. As a result, finding sturdy classes of generative models (like stochastic block models^171^ or Gaussian models) that can effectively classify omics data is difficult. Moreover, biological sequencing and networks are intricate encodings of bioactivities, but few systematic ways exist to read these encodings in the same manner that humans understand semantics or context from written language. These disparities make it difficult to gain insights from data exploration and construct and test ideas. Engineering biology entails the problem of knowing about a black box entity, in which we can witness input and output but have little knowledge of the system’s inner workings. Given the immense combinatorial parameter space in which these biological systems work, AI applications that strategically and effectively organize experiments to explore and scrutinize biological systems for the generation and verification of hypotheses present an enormous need and opportunities in this sector.^172,173^

Finally, many prominent AI technological solutions do not account for uncertainties and lack effective mechanisms for controlling errors in the face of input perturbation. Given the inherently stochastic nature and chaos in the natural (biological) systems I am attempting to engineer, this fundamental gap is crucial in the synthetic biology realm.

#### Metrics/Evaluation-Based Challenges

Traditional AI evaluation metrics relying on prognosis and accuracy are inadequate for synthetic biology applications. Metrics like 2P for regression analysis or precision for classifying models do not consider the complexity of the underlying biosystems I am attempting to represent. In this subject, additional metrics that evaluate the extent to which a model can reveal the internal workings of a biological system and preserve a preexisting knowledge base are equally significant. To that aim, AI systems that integrate the principles of transparency and interpretability are crucial in promoting iterative and transdisciplinary research. Furthermore, the ability to accurately measure uncertainty necessitates the creative development of innovative metrics to assess the efficacy of these approaches.

Metrics are also required for proper experimental design. Model evaluation and validation in synthetic biology may necessitate further experiments, necessitating additional resources. Even a minor error or misclassification can have a significant effect on the research goal. To depict the actual impact of a misclassification, these costs should be included in objective functions or the assessments of AI algorithms.

### Sociological Challenges

In harnessing AI to benefit synthetic biology, sociological barriers may be more challenging to conquer than technical ones (and vice versa). Many difficulties, in our opinion, originate from an absence of coordination and comprehension among the many varied cultures involved. While some projects have begun to address these issues, it is worth noting that recurrent themes remain troublesome in industry and academia.

#### Genesis of Sociological Challenges

Sociological challenges stem from the necessity of blending expertise from two distinct groups: bench scientists and computational scientists. Bench and computational scientists receive quite different training. Computing scientists are trained to focus on abstractions, to be enthralled by automation and computational efficiency, and to embrace disruptive techniques. They are naturally inclined toward task specialization and seek ways to delegate repetitive duties to an automated computing device. Bench researchers are practical, have been trained to work with tactile observations, and favor explainable analyses to precisely characterize an experiment’s outcome.

The bench and computational worlds have distinct cultures, which are reflected not only in how they handle problems but also in which problems they believe are worth solving. For example, there is a constant tension between the amount of work spent to establish the infrastructure that enables broad research and the amount of effort devoted to studying a specific research subject. The computational researcher prefers to provide a trustable infrastructure that can be relied on for a range of tasks (for instance, an automated stream for strain development or a centrally controlled database gathering all pertinent information), whereas bench researchers typically concentrate on the end goal (for instance, generating a desired molecule in commercially valuable amounts), even though that means they rely on bespoke strategies that can only be valid in that particular instance. Computational researchers want to create mathematical models that describe and predict the activities of biological systems, while bench researchers prefer to generate qualitative ideas and test them empirically as soon as feasible (at least while experimenting with microorganisms, as those investigations can be finalized rapidly: 3–5 days). Besides that, computational scientists are often only enthusiastic and invigorated by noble, blue-sky goals such as bioengineering lifeforms to terraform Mars, trying to write a life compiler capable of creating DNA to accomplish an optimum setting, reengineering trees to embrace contour, bioengineering dragons in actual situations, or AIs looking to replace researchers. Bench researchers perceive these grandiose ambitions as “hype”, are burned by prior examples of computational types overpromising and underdelivering, and would rather only explore goals that can be achieved with existing technology.

#### Taking on Sociological Challenges

The remedy to the social challenges is to value multidisciplinary teams and needs. To be sure, creating this inclusive atmosphere may be easier in a corporation (where the team succeeds or sinks together) than in an academic setting (where a graduate or postdoc pursues research just to get some first-author publications to get a job, without collaboration with other disciplines). Developing cross-training courses where computer researchers are trained in experimental research and bench researchers are trained in programming and ML is one viable path for this integration. Finally, both groups provide something valuable, distinct, and significant to the board. The sooner everyone involved understands this, the faster synthetic biology can progress. In the long run, university curricula that integrate biological and bioengineering with automation and arithmetic are required. Though several projects are already ongoing, they are only a drop in the ocean of the required manpower.

## Latest DL Methods to Address the Challenges and Outlook

In this section I have presented the latest DL methods and perspectives for addressing the above-mentioned challenges.

### Pretrained Self-Supervised Models for Alleviating the Challenge of Data Insufficiency

Pretrained models can achieve state-of-the-art performance on various natural language processing (NLP) tasks. Pretrained models like BERT,^174^ GPT,^175−177^ and others are trained on massive corpora of text data. They are exposed to a vast amount of diverse language patterns, which helps them learn rich and contextualized representations of words and sentences. The pretraining process in these models involves self-supervised learning tasks, such as masked language modeling and causal language modeling. These tasks require the model to predict masked or next tokens, forcing it to learn contextual relationships within the text. Pretrained models exhibit strong transfer learning capabilities. They can be fine-tuned on specific downstream tasks with relatively small amounts of labeled data. The pretrained knowledge about language and context, captured during pretraining, acts as a powerful template for these downstream tasks. Pretrained models can be updated and adapted to new data without retraining from scratch. This ability to perform continual learning allows them to stay relevant and adapt to changing data distributions.

The pretrained models for processing biological sequences, particularly protein and DNA sequences, are inspired by transformer-based architectures, like BERT, but adapted to handle the unique characteristics of biological data. For instance, Rives et al.^178^ developed ESM-1b (Evolutionary Scale Modeling) which is a 33-layer Transformer model with 650 million parameters developed for protein sequence modeling. It is trained using BERT-like masked language modeling on a large data set of 250 million protein patterns from Uniref 50,^179^ which contains clusters of patterns with 50% similarity in the UniProt Archive. By fine-tuning small data sets, downstream classifiers achieve strong performance on tasks like predicting protein secondary structure and contact map. DNABERT^180^ is developed for DNA sequence modeling and is based on a 12-layer BERT-base^174^ Transformer model with 110 million parameters. It is pretrained on the k-mer portrayal of the human genome using masked language modeling, where the human genome is tokenized into k-mers. DNABERT exhibits similar or superior performance compared to other models on various sequence classification tasks, including promoter recognition, functional genetic variant classification, splice site prediction, and TF binding site prediction. Additionally, DNABERT demonstrates cross-species transfer learning capability by predicting mouse TF binding sites. The MSA Transformer^181^ (Multiple Sequence Alignment Transformer) extends the transformer model to handle MSAs of amino acid sequences. By leveraging contextual information within individual sequences and across homologous sequences, the MSA Transformer shows even better performance on downstream tasks like protein secondary structure and contact map prediction compared to ESM-1b.

Overall, the use of language modeling as a pretraining objective enables pretrained models to efficiently learn from vast amounts of diverse and unlabeled biological sequence data. Language modeling can create context-dependent representations which can be used to improve performance on various biological prediction tasks. For instance, LM of proteins can develop context-dependent representations, and these representations can be employed to improve the performance of several protein prediction tasks. Moreover, with the understanding of protein likelihood, a researcher can filter, autocomplete, and generate new proteins. However, for this goal, language models should be capable of generating high contextual understanding related to protein sequencing from all domains of interest.

This approach has significantly advanced the field of bioinformatics and computational biology, providing powerful tools for biological sequence analysis and prediction tasks.

### Few-Shot or/and Meta-Learning Mechanisms Result in Data Efficient DL Models

The challenge of data insufficiency can also be tackled by developing a DL model that uses data efficiently. Meta-learning is useful in scenarios with limited labeled data, few-shot or one-shot learning settings, and tasks with high variability. DeeReCT-TSS^182^ is a deep learning model designed for predicting transcription start sites (TSS) in different cell types. The authors applied a gradient-based meta-learning algorithm called Reptile to facilitate fast adaptation of the TSS prediction model to multiple cell types. The use of Reptile allowed the model to quickly adapt to new cell types with minimal labeled data from each cell type. Mutual information maximization meta-learning (MIMML)^183^ is a novel meta-learning framework designed specifically for predicting the function of bioactive peptide. It leverages the Prototypical Network, which is a few-shot learning approach used for classification tasks, to perform predictions for a total of 16 different peptide functions.

### Benefit Modeling by Including Structural Information

The sequence-only models are limited to explicitly consider transacting factors. Such factors usually depend on protein–protein^184−187^ and protein–nucleic acid interactions at a molecular level. Hence, to accurately model these factors in gene regulation, it is essential to incorporate structural information from both cis-acting and trans-acting counterparts. Indeed, recent breakthroughs in protein structure prediction, particularly the development of AlphaFold2, have significantly advanced our understanding of protein structures.^188^ AlphaFold2, developed by DeepMind, demonstrated remarkable accuracy in predicting protein 3D structures during the Critical Assessment of Structure Prediction (CASP) competition. This breakthrough has enriched our resource for protein structures and has the potential to transform the field of structural biology. Additionally, progress has been made in predicting secondary structures of RNA and 3D structures of the genome.^189−192^ The availability of accurate structural information for proteins, the genome, and RNA opens new possibilities for systematically incorporating this structural information into deep-learning models for gene regulation. By integrating structural data with deep-learning approaches, researchers can create more comprehensive and precise models of gene regulation at the molecular level.

Incorporating structural information from protein 3D structures into DL models has the potential to enhance our understanding of complex biological processes and regulatory networks. By leveraging the insights gained from MaSIF^193^ and dMaSIF,^194^ researchers can explore new avenues for modeling gene regulation, protein–protein interactions, protein–ligand interactions, and other molecular interactions, ultimately leading to advancements in proteomics and systems biology. Indeed, NucleicNet^195^ is an excellent example of a transcriptomic-level model that incorporates structural information to predict binding specificities of RNA-binding proteins (RBPs). By representing the binding 3-D structure of protein as a 3-D grid with physicochemical possessions and using a CNN with residual connections, NucleicNet achieves accurate predictions of RBP binding preferences for different RNA constituents.

### Multiomic Model Development

Indeed, biologists often employ multiple experimental techniques to strengthen the validity and reliability of their findings. By using different methods, they can cross-validate their results and reduce the likelihood of errors or biases. The work by Chaudhary et al.^196^ is an excellent example of utilizing multiomics data and DL techniques for the prediction of survival of patients with hepatocellular carcinoma (HCC). The model was trained employing 230 samples from TCGA (The Cancer Genome Atlas) with RNA-seq data, DNA methylation profiles, and microRNA-seq data. The process of autoencoder-based dimensionality reduction,^197^ feature selection, and concatenation helps to mitigate the challenges posed by high-dimensional omics data and enhances the model’s ability to capture relevant biological signals. The integration of multiomics data with concepts from multimodal machine learning^198^ holds great potential for driving innovations in precision medicine and personalized healthcare.

The MOMA^199^ (Multi-Omics Model and Analytics) model is a sophisticated approach used to predict multiomics quantities of *E*. *coli* based on different growth conditions. MOMA combines RNN-based DL and LASSO (Least Absolute Shrinkage and Selection Operator) regression to achieve its predictions. The model acquires a layer-by-layer process to predict proteomic, transcriptomic, metabolomic, phenomic, and fluxomic quantities sequentially, while considering the influence of quantities from previous omics layers on the current prediction. The Deep Structured Phenotype Network (DSPN)^200^ is a powerful model designed to predict brain phenotypes using several functional genomic data modalities. The DSPN utilizes a hierarchical conditional deep Boltzmann machine (DBM) architecture^201^ for its predictions. This approach allows the model to capture complex interactions and dependencies between different genomic data types and their relationships to brain phenotypes.

### Usage of Single-Cell Profiles

The advanced single-cell omics technologies have greatly expanded our understanding of cellular diversity, developmental processes, disease mechanisms, and the complexity of various tissues and organs. They continue to be refined and applied in diverse fields, from developmental biology and immunology to cancer research and regenerative medicine. Single-cell ATAC-seq (scATAC-seq)^202,203^ for chromatin accessibility profiling, single-cell RNA-seq (scRNA-seq) for gene expression level profiling, single-cell reduced representation bisulfite sequencing (scRRBS-seq)^204^ for methylation profiling, single-cell bisulfite sequencing (scBS-seq),^205^ Smartseq^206^ for full-length transcriptome profiling, and single-cell Ch IP-seq (scChIP-seq)^207^ for protein–DNA binding profiling are some of the key single-cell omics profiling technologies that have seen substantial improvements. Current DL-based gene regulation models use single-cell profiles basically in two different ways. One operates at the genuine single-cell level, while the other operates at the pseudobulk level.

Current DL-based gene regulation architectures generally employ single-cell profiles in two divergent ways. The first works at the pseudobulk level. The model assembles single-cell assessments of each cell cluster into a single profile. The assembled pseudobulk profiles are then used by the model in a manner like how bulk omics profiles are used. Regardless of loss of information during aggregation, the employment of pseudobulk profiles still has an advantage over real bulk omics profiles as they depict evaluations from pure cell types without interference from others. The utilization of pseudobulk profiles in the context of single-cell omics analysis has advantages over real bulk omics profiles, despite the information loss that occurs during the aggregation process. The study conducted by Cusanovich et al.^208^ involved single-cell ATAC sequencing (scATAC-seq) on around 100,000 somatic cells of mature mice. The researchers aimed to predict chromatin accessibility for each identified cell type using a multitask learning approach based on the Basset architecture. They trained the model based on aggregated pseudobulk profiles inside each cell cluster. Recently, based on DeepMEL, Janssens et al. presented DeepFlyBrain model for predicting chromatin coaccessible areas in the Drosophila brain.^209^

DeepCpG^210^ is a deep learning model designed for imputing methylation status in low-coverage single-cell DNA methylation profiles. The model was trained on scBS-seq (single-cell bisulfite sequencing) and scRRBS-seq (single-cell reduced representation bisulfite sequencing) data from multiple mouse and human tissues. The model architecture combines CNNs with bidirectional Gated Recurrent Units (GRUs). SCALE^211^ is a DL model designed for imputing low-coverage single-cell ATAC sequencing (scATAC-seq) profiles. The model is based on a combination of variational autoencoder (VAE) and Gaussian mixture models (GMMs). It is specifically tailored to address the challenge of handling sparse and missing data in scATAC-seq profiles. DL approaches have also shown promising results in making inferences on gene regulation networks employing single-cell RNA seque (scRNA-seq) data. CNNC^212^ is one such example of a DL model designed for inferring the causality between two genes in a gene regulatory network. Many latest methods and perspectives to overcome the challenges have been summarized in table format^213−245^ (Table S1).

## Conclusions and Future Perspectives

The widespread adoption of Next Generation Sequencing^246^ has facilitated the generation of enormous data sets, but they are constrained to evaluations in chromatin accessibility, genomic data, and transcriptome profiles. Other biological scale assessments, including metabolomics proteomics, are gradually catching up to the data quantities generated by NGS-based approaches. Biological diversity is often difficult to manage since it is caused by random mutations that occur throughout generations. This inconsistency is not usually handled and can induce noise in quantified biological data. This repetitive noise, combined with transcriptomic variability, has an influence on data reproducibility and can degrade model fitting quality. Techniques like denoising filters can help to overcome this barrier.^247,248^ Unsupervised learning can be vital in determining hidden relationships among elements in intricated high-dimensional biological data.

In synthetic biology, ML algorithms already play a crucial role in supporting the Learn part of the DBTL^249,250^ cycle. By learning more systematically from the training data set of newly generated mutants, these models can reduce the turnover time of each DBTL cycle by obtaining additional expertise for every round and creating better trials sequentially. The use of automation in experimental biology can expedite the emergence of fully automated DBTL cycles which are autonomous of human involvement. ML has apparent uses in standard optimization tasks for driving strains toward desired targets, but developing framework modeling to achieve a basic biological system perspective is a less evident challenge. A union of machine learning, mechanistic models, and automated biofoundries will almost certainly result in some of the most significant discoveries in synthetic biology shortly.

The combination of synthetic biology with deep learning research promises the development of new sequences and constructs, data analysis automation, optimal experimental designs, and multiple other applications. The DL study emphasizes that basic models can have significant advantages. Owing to several parameters and the complexity of the frameworks involved, DL models can practically be black boxes, decreasing the model’s interpretability.

Before moving on to DL models, it is often vital to experiment with basic ML approaches to better understand their performance. Sample et al.^90^ examined a linear regression model on the 5′ UTR data set, which provided a good point of comparison to their CNN-based findings. It will also be beneficial to comprehend the trade-offs between efficiency and complexity for diverse applications, and research into this area is likely to be beneficial. For example, Nikolados et al.^251^ evaluated the ability of models of complexity to determine protein production from DNA sequence. Eventually, the amount of available data also has a significant impact on whether DL strategies are viable because deep models need large training sets. Many of the latest DL methods are efficient to overcome the challenges of ML/DL in biosystems and have been summarized in table format (Table S1).

Overall, ML and DL strategies have had a considerable impact on the synthetic biology field, and I foresee significant progress in this area in the future. In this review, I attempted to present an overview of ML and DL methodologies and applications in synthetic biology. I have also addressed the challenges and opportunities for dealing with biological data sets, with the purpose of assisting professionals in incorporating ML and DL approaches, and insights into their arsenal.

## Supporting Information Available

Table S1 containing the latest methods and perspectives to overcome the challenges of conventional ML/DL methods (PDF)
