# Supplementary material for: Machine Learning and Deep Learning in Synthetic Biology: Key Architectures, Applications, and Challenges
Source: ACS Omega. 2024 Feb 19;9(9):9921–45. doi: 10.1021/acsomega.3c05913 (PMC10918679; doi:10.1021/acsomega.3c05913)
Supplement: Supplementary file 1 — ao3c05913_si_001.pdf [file ao3c05913_si_001.pdf]

Supporting Information

Machine Learning and Deep Learning in Synthetic Biology: Key Architectures, Applications, and Challenges

Manoj Kumar Goshisht*

Department of Chemistry, Natural and Applied Sciences, University of Wisconsin-Green Bay, Green Bay, Wisconsin 54311-7001, United States

Email: kumarm@uwgb.edu , mkgh07@gmail.com

Table S1. Latest methods and perspectives to overcome the challenges of conventional ML/DL methods.

Method and Year	Datasets	Model	Functionalities
Self-supervised pre-trained models			
ESM-1b Transformer[178] (2020)	Trained on 250 million protein sequences from UniProt Archive[213] and collected by Uniref. 50.	Transformer architecture (33 layers and 650M parameters)	(i) performs masked language modeling. ii) secondary structure prediction (iii) contact map prediction
MSA Transformer[181] (2021)	260 M (million) MSAs from UniProt controlled by UniClust30[214]	Transformer adapted to MSA (12 layers, 100 M parameters)	(i) protein masked language modeling. (ii) prediction of supervised and unsupervised contact map (iii) secondary structure prediction.
DNABERT[180] (2021)	k-mer representation of human genome	BERT-base[174] Transformer architecture (12 layers with 110M parameters)	(1) DNA masked language modeling. (2) strong performance on (i) prediction of TF binding sites, (ii) promoter recognition, (iii) splice sites prediction, (iv) cross species transfer learning, (v) functional genetic variants classification,

Few-shot or/and meta- learning			
vMIMML[183] (2022)	starPepDB[215] BIOPEP-UWM[216]	Prototypical Network[217] performing meta-learning classification (few-shot learning)	To predict functions of bioactive peptide
DeeReCT-TSS[182] (2021)	FANTOM5[218]	Reptile algorithm[219] for meta-learning	Permits fast adaptation to new tissue types
Structural information incorporation			
MaSIF[193] (2020)	Protein Data Bank	Geodesic CNNs[220]	(i) **MaSIFligand** for protein pocket classification. (ii) **MaSIF-site** for protein interface prediction. (iii) **MaSIF-search** for protein-protein interaction (PPI) search
NucleicNet[195] (2019)	483 protein-RNA complexes from the protein data bank[221] and de-duplicated to 158 ribonucleoprotein structures	Convolutional Neural Network with ResNet-like architecture	To predict RNA-protein binding based on structure.
dMaSIF[222] (2021)	Protein Data Bank	Quasi-geodesic convolution on point cloud representation of protein surfaces	(i) To predict protein interfaces (ii) PPIs search
Multiomics models			
DSPN[200] (2018)	The study assembled the dataset resource ''PsychENCODE,'' that includes broad functional genomic data (Hi-C profiles, genotype, Chromatin, and bulk transcriptome) of the brain of 1,866 individuals	Conditional DBM (deep Boltzmann machine)[201]	(i) To predict "intermediate molecular phenotypes" (ii) To predict brain phenotypes from several functional genomics techniques
Chaudhary *et al.*[196] (2018)	230 specimens from TCGA with microRNA-seq data, RNA-seq data, and DNA methylation profiles	Dimensionality reduction based on autoencoder,[197] feature selection, and	(i) To cluster patients into divergent survival groups,

		multi-omics data integration.	(ii) survival-related autoencoder attributes have substantiated predictive<br><br>rendition on independent datasets
deepManReg[223]<br><br>(2022)	the Patch-seq[224] multi-omics<br><br>transcriptomic and electrophysiological<br><br>data for neuron phenotype<br><br>classification[225]	DNN accompanied by manifold alignment[226]	multi-modal alignment of multi-omics<br><br>data<br><br>Multi-modal alignment of electrophysiological and transcriptomic data in the multi-omics experiment[224]
Single-cell profile utilization			
Pseudobulk level			
DeepFlyBrain[209] (2022)	scATAC-seq profiling of Drosophila brain cells (240919 cells)	CNN + LSTM architecture employed in DeepMEL	To predict co-accessible sites in three cell subtypes: Glia, Kenyon cell, and T<br><br>neurons
Single-cell level			
DeepCpG[200]<br><br>(2017)	(i) Smallwood *et al.*[205], single-cell bisulfite sequencing (scBS-seq) for muse tissue.<br><br>(ii) Hou *et al.*,[227] single-cell reduced representation bisulfite sequencing (scRRBS-seq) for human and mouse tissue	CNN + bidirectional GRU architecture	To impute methylation status at the single-cell level
SCALE[215,211]<br><br>(2019)	(i) GM12878/HEK293T dataset from[198]<br><br>(ii) acute myeloid leukemia dataset from[228]<br><br>(iii) In silico dataset[202,229]<br><br>(iv mixture of mouse splenocyte dataset[230]	variational autoencoder (VAE)[233] with Gaussian mixture model	(i) Imputation for scATAC-seq data<br><br>(ii) Batch effect removal and clustering

	(v) In silico mixture of scATACseq experiments of six cell lines<br><br>(vi) P56 mouse forebrain dataset[231]<br><br>(vii) mixture of tumor epithelial cells and tumor-infiltrating immune cells<br><br>(viii) breast tumor dataset[232]		
CNNC[212]<br><br>(2019)	(i) mouse scRNA-seq dataset[234]<br><br>(ii) 43261 expression profiles from more than 500 divergent scRNAseq studies<br><br>(iii) The GTRD database[235] for mESC ChIP-seq peak regions<br><br>(iv) Reactome pathway[236] data.	CNN	(i) To predict interaction between two genes<br><br>(ii) TF target gene prediction,<br><br>(iii) To predict causality inference between genes<br><br>(iii) To predict disease related genes
scGNN[237]<br><br>(2021)	scRNA-seq datasets:<br><br>(i) the Klein data (GEO: GSE65525)<br><br>(ii) the Chung data (GEO: GSE75688)<br><br>(iii) the AD case data (GEO: GSE138852)<br><br>(iv) the Zeisel data (GEO: GSE60361)	Graph Neural Network (GNN)-based autoencoder	(i) scRNA-seq data imputation<br><br>(ii) Clustering

scFAN[238] (2020)	ENCODE GM12878, H1-ESC, K562 TF binding profiles	Three-layer CNN	To infer TF binding activity of scATACseq employing bulk data trained TF binding model.
scTenifoldKnk[239] (2022)	Gene knockout (KO) datasets: (i) Trem2[240] (ii) Hnf4a and Hnf4g[241] (iii) Nkx2-1[242]	Before and after KO Quasi-manifold alignment[243]	To predict virtual gene knockout
scBasset[244] (2022)	FACS-sorted scATAC-seq of cells with hematopoietic differentiation[245] 10x multiome (scATAC-Seq + scRNA-seq) of PBMCs	6-layer CNN	(i) To impute scATAC-seq profile. (ii) Cell clustering and de-noising
